# Acceptance Testing Methodology for the Production of Standard Reference Material 2806: Medium Test Dust in Hydraulic Fluid

**DOI:** 10.6028/jres.126.044

**Published:** 2022-02-24

**Authors:** Robert Fletcher, James Filliben, Nicholas Ritchie, Nicolas Petillon

**Affiliations:** 1 National Institute of Standards and Technology, Gaithersburg, MD 20899, USA; 2 IFTS, Institut de la Filtration et des Techniques Séparatives, International Filter Testing Service, 3 rue MARCEL PAGNOL, 47510 Foulayronnes France

**Keywords:** experimental design, five-step method, hydraulic fluid, medium test dust, optical particle counting, standard reference material, statistical test, testing

## Abstract

Standard Reference Material (SRM) 2806: Medium Test Dust in Hydraulic Fluid represents a series of reference materials certified by the National Institute of Standards and Technology (NIST) used to calibrate liquid-borne optical (or automatic) particle counters applied in a wide range of industrial, aerospace, and military applications. The series, including SRM 2806b, and SRM 2806d, was manufactured for NIST by IFTS, Institut de la Filtration et des Techniques Séparatives International Filter Testing Services, in France. An important factor for the acceptance of the material for certification was the degree of bottle-to-bottle homogeneity, which was evaluated by both IFTS and NIST. A statistical graphics methodology was developed that provided immediate visual as well as quantitative statistical metrics with which to characterize the SRM. This NIST-developed approach was used in four studies to assess the homogeneity of the material during both its production stage and its finished bottled-product stage. IFTS performed measurements using an optical particle counter for on-line quality assurance and sampled 40 bottles of the finished 400 bottle series to determine homogeneity from the particle size distribution. NIST also determined the particle size distribution of the finished material and performed microscopy to look for possible contaminant material in the suspension. An accelerated aging experiment was conducted on both materials (2806b and 2806d) to verify their stability.

## 1 Introduction

Standard Reference Material (SRM) 2806: Medium Test Dust in Hydraulic Fluid is intended for use as a reference material for calibrating liquid-borne particle sizing instruments, specifically, optical particle counters. This reference material is widely used for the important reason that it is estimated that about 80% of all hydraulic failures are due to particulate contamination in the hydraulic fluid [1]. Therefore, contamination control is critical. Equipment failure is costly in terms of repairs, lost productivity, safety, and waste generation. Particle counters are routinely used to monitor fluid cleanliness in operating systems, to assess filter performance, and to evaluate the cleanliness of equipment coming off production lines as well as of individual components. This SRM supports the hydraulic contamination control delineated by International Organization for Standardization (ISO) Technical Committee 131/SC6 WG1: the document ISO 11171:2020 [2] and other associated documents under the committee’s purview. SRM 2806 is a particle counter calibrant for liquid suspensions and a required reference material for ISO 11171:2016 and 11171:2020—Hydraulic fluid power—Calibration of automatic particle counters for liquids [2]. Industries impacted by SRM 2806 include the hydraulics, aerospace, automotive, shipping, petroleum, lubricant and gas industries, power generation, filter manufacturers, and the military.

For most materials to become certified by the National Institute of Standards and Technology (NIST), they must qualify based on a set of measurement and statistical criteria. Two such criteria for a NIST-certified reference material are that the material be (1) homogeneous and (2) stable with respect to composition or physical characteristics. The homogeneity, in this case, is established by comparing the particle size distribution over a select number of sample bottles. The stability can be verified by subjecting the candidate reference material to an accelerated aging procedure and assessing whether there are physical changes in the material relative to the unaged materials. Possible contaminates are investigated using scanning electron microscopy (SEM) and optical particle counter measurements. The two-step procedure described here represents the first phase of the certification process. Without establishing homogeneity and stability, the material would not be further characterized.

With the objective of making a new candidate material, a collaboration was formed between NIST and the Institut de la Filtration et des Techniques Séparatives (IFTS), International Filter Testing Services, Foulayronnes, France, to determine the quality and suitability of any new candidate materials for use as SRM 2806 and to document the acceptance procedure of any new material. In this report, we describe how material quality was defined and quantified by NIST-provided performance metrics, and how this led to a structured sequential improvement process for the candidate material. Without the quality metrics, it is unlikely that this SRM could have been produced.

The prospective material for SRM 2806 was composed of MIL-PRF-5606 hydraulic fluid augmented with a trace amount of an SAE 5-80 medium test dust (a quartz mineral dust) [3].^1^1 Certain commercial equipment, instruments, or materials are identified in this report to specify adequately the experimental procedure. Such identification does not imply recommendation or endorsement by the NIST, nor does it imply that the materials or equipment identified are necessarily the best available for the purpose. The polydisperse medium test dust came from the same batch of dry dust, 4390C, that has been used to make both past SRM 2806 and reference material (RM) 8631. The candidate material for SRM 2806: Medium Test Dust in Hydraulic Fluid was manufactured by IFTS. IFTS performed measurements using optical particle counters for on-line quality assurance (described below) and sampled 40 bottles of the finished product to determine the homogeneity by measuring the particle size distributions from these samples.

NIST received (from IFTS) approximately 32 bottles of the prospective SRM 2806 material that was selectively sampled from the 400-bottle global set to provide representative bottles from each quartile of the production phase (1 to 100, 101 to 200, 201 to 300, and 301 to 400). One sampling criterion was that bottle pairs were selected to be adjacent (*i.e.*, consecutive) in the production process. This adjacency would assist in discriminating between instrument performance issues and material inhomogeneity issues.

NIST determined the particle size distribution of the finished material and performed electron microscopy to look for possible contaminate material in the suspension. Measurements from IFTS and NIST were in sufficiently good agreement (as per the specification document in the Appendix) to consider the candidate material as acceptable for the certification process. The present paper gives an overview of the general method used to qualify a new batch of candidate 2806 reference material and presents specific statistical tests, characterization procedures, and results for both SRM 2806b (June 2014) and SRM 2806d (March 2021).

## 2 Material Production

**Fig. 1:**
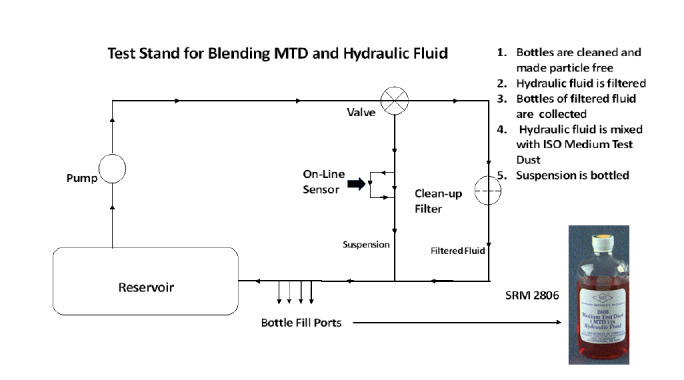
Schematic of the IFTS process for manufacturing SRM 2806. The test stand system is equipped with an on-line particle sensor and ports to fill indivdual bottles with the hydraulic fluid medium–test dust (MTD) suspension. The purpose is to provide bottle-to-bottle homogeneity with regard to the cumulative particle concentration.

The medium test dust RM 8631b and the MIL-PRF-5606 hydraulic fluid were blended together by IFTS using a test stand that first filtered the hydraulic fluid to provide a near-particle-free base material. Then, medium test dust was added, and the dust was mixed with the fluid by recirculation. A schematic of the process (but not the actual test stand) is shown in Fig. 1. The bottle-fill process took a nominal 2 h duration. It is well known that mechanical mixing is a challenging process, especially when the goal is to achieve homogeneity.

IFTS performed on-line particle counting to ensure mixing was taking place and a high degree of homogeneity was attained in the test stand during the mixing process. These on-line readings were conducted and recorded before the bottle-filling process commenced. NIST provided a statistical diagnostic tool (discussed in further detail in this document) that helped IFTS uncover possible problems in prospective batches of material; this tool was applied dynamically to on-line measurements during the IFTS material mixing process and before the laborious and expensive bottling process. After arriving at a suitable IFTS mixture, 400 filled bottles of the material were produced, with select bottles tested by the manufacturer and requested bottles shipped to NIST, where subsequent bottle-to-bottle homogeneity testing was carried out. The bottle-to-bottle measurements were the most important indicator because they provided a representation of the variation found in the finished material.

Analysis at NIST was conducted using two techniques: (1) automatic optical particle counting using an HRLD 600 sensor equipped with an auto-sampler operating in the volumetric mode and (2) microscopy using an automated SEM commercially known as the TESCAN (TESCAN MIRA3, TESCAN, Brno, Czech Republic) automated with the SEMantics extension to NIST DTSA-II [4]. Image analysis was conducted using the NIST-developed Lispix [5].

The following steps were taken to assure the quality of the SRM:

(1) Homogeneity testing was conducted to intercompare the manufacturer’s and NIST’s measurements from optical particle sensors. Experiments indicated that the manufacturer’s results and NIST’s results agreed, and (based on homogeneity) the new material was of high quality and was acceptable.
(2) SEM survey analysis was conducted to test for contaminate particles and to perform image analysis to determine cumulative particle size concentrations. These measurements indicated that there was no obvious contaminate particle material in the candidate SRM.
(3) NIST conducted accelerated aging testing to verify the stability of the new candidate material as a function of time for SRM 2806, and results showed that the material did not change due to heating or cooling over 90 d.
(4) NIST applied a five-step statistical analysis to verify the quality of the material based on the optical particle count data.

## 3 Physical Tests of Hydraulic Fluid Suspension

Each batch of hydraulic fluid medium–test dust suspension submitted to NIST was tested using four methods: (1) NIST-designed statistical bottle sampling to facilitate optical particle counting; (2) automatic optical particle counting to verify the particle concentration; (3) accelerated aging test to verify the stability of the hydraulic fluid; and (4) SEM analysis using an automated TESCAN instrument to look for particle contaminates.

### 3.1 Bottle Sampling

The bottles analyzed by IFTS and by NIST were chosen using “design of experiments” statistical procedures provided by NIST. Bottles were taken from the global collection of 400 bottles as specified in the Appendix of this document, and the sampling procedure was designed to sample the material over the complete production sequence, but randomized for possible systematic problems like filling nozzle anomalies or circulation flow rate variation. From specifications given in the Appendix: The manufacturer analyzed one, randomly-chosen bottle out of each 10 bottles produced for within-batch homogeneity as described in ISO 11171 clauses F.3 through F.5, except that only 40 bottle samples were analyzed (by IFTS) in total. For example, one randomly chosen bottle was chosen from the first 10 produced bottles (bottles 1–10), one from the second group of 10 bottles (bottles 11–20), one from the third group of 10 bottles (bottles 21–30), *etc*.

NIST tested eight bottles for within-batch homogeneity using an extinction automatic optical particle counter calibrated to the previously certified SRM 2806. The eight bottles consisted of two bottles taken from each quartile of the production labeled assignment. The data were compared to analogous data from IFTS. The eight bottles were selected such that each pair was composed according to the Appendix sampling procedure from the 400 bottles. In the case of candidate reference material 2806b, the sample bottles analyzed by NIST were the following (not in this order): 27, 28, then 171, 172, then 249, 250, and finally 361, 362. The NIST bottles were always analyzed in a randomized sequence to decouple possible manufacturing bias from optical particle counter influences.

### 3.2 Automatic Optical Particle Count Data

Each bottle of candidate SRM 2806x analyzed was thoroughly mixed (according to recommended practice of ISO 11171:2020) to suspend and disperse the particles to form a uniform particle suspension in the hydraulic fluid. Each closed bottle of SRM 2806x was shaken by hand or laboratory shaker for approximately 1 min. The contents of the bottle were dispersed using an ultrasonic bath with minimum intensity of 3 000 W/m^2^ for 1 min. Then, the bottle was mechanically shaken for a minimum of 3 min on a commercial paint or laboratory shaker. The bottle was briefly treated to ultrasonication or evacuated to remove air bubbles from the suspension and then was introduced immediately into the liquid automatic particle counter. Both IFTS and NIST analyzed three 80 mL aliquots of each bottle of material using an instrument with a light blockage or extinction sensor covering the particle size range of 4 μm to 100 μm. The sensors were calibrated using NIST SRM 2806 (the previously certified SRM) to provide a common calibration for both particle sensors and serve as a basis for determining the particle number distribution of candidate SRM 2806x. This single calibration was used throughout by both laboratories to standardize the measurements. In subsequent studies to be more in tune with the typical analysis procedure, five 10 mL samples will be analyzed instead of three 80 mL aliquots, and a sensor that detects 4 μm (c) and greater will be used.

### 3.3 Accelerated Aging Test

Two bottles of candidate SRM 2806b and SRM 2806d materials were sealed and placed in a 75 °C oven, and two bottles from each batch were placed in a refrigerator at approximately 4 °C. The materials were monitored for 90 d to see if this hydraulic fluid with its additives degraded. All four bottles were previously unopened, and all contained ISO Medium Test Dust. For SRM 2806b, both the candidate reference material and the clean filtered hydraulic fluid were subjected to the aging tests. For candidate SRM 2806d, only the standard material was used, and the pure oil was not subjected to either thermal challenge.

During the thermal tests, the bottles were periodically examined visually while at their respective temperatures either (75 °C and 4 °C), *i.e.*, not cooled/warmed to laboratory temperatures. Visually, they always appeared clear. After terminating the thermal exposure at 90 d, the bottles were then allowed to come to room temperature of ~22 °C. About 50 mL of oil from each sample was filtered using a clean separate 0.4 μm pore polycarbonate filter (track etched filter, 10 μm thick). The filtered sample was washed using filtered reagent-grade heptane, evacuated for 48 h, gold coated with an Ar plasma sputter coater, and examined by SEM. SEM and optical particle analysis indicated no evidence of fluid degradation. No extraneous particles were observed, and only the medium test dust particles that were added to make the reference material were detected.

All bottles were analyzed with the optical particle counter calibrated to the same scale used to qualify the material. The results indicated no additional particles were formed in the hydraulic fluid during the accelerated aging process. The particle size distributions were found to be within the measurement uncertainty of the bottles not subjected to the temperature challenges.

### 3.4 SEM

All bottles of candidate SRM 2806 subjected to microscopy analysis were hand shaken, sonicated for 1 min, and shaken again by a mechanical shaker for 3 min before immediately sampling. Approximately 50 mL aliquots of the hydraulic fluid test dust suspension were filtered using 37 mm diameter, 0.4 μm pore size polycarbonate filters. The filtered samples were washed three to four times each with a clean, prefiltered heptane solvent to remove the oil residue. The filters were evacuated over 48 h to remove any volatile organic vapors. Filters were gold coated with 9 nm to 18 nm thick gold delivered by an Ar plasma coater and were mounted on a metal stage and analyzed using the TESCAN MIRA 3 SEM.

SEM images of filtered particles were collected from each of two bottles of candidate SRM 2806b and two bottles of ultrafiltered hydraulic fluid, the starting fluid used to make the SRM. The purpose was to verify the cleanliness of the hydraulic fluid samples, make sure no extraneous particle formation occurred in the hydraulic fluid, and verify that the micrograph images of the medium test particles could be image processed.

**Fig. 2:**
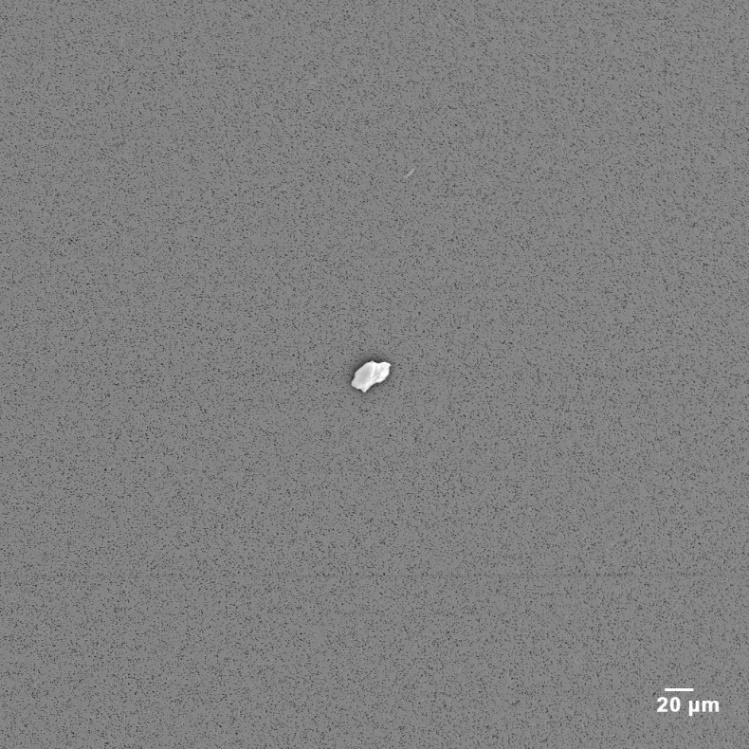
SEM micrograph of filtered clean oil with image field of 0.5 mm.

Figure 2 shows a typical SEM micrograph of the clean filtered oil at 0.5 mm full-field magnification. A single trace particle was found after searching the filter to aid in focusing the electron beam and thus imaging the field. There are virtually no contaminate particles in either bottle of filtered clean fluid material.

**Fig. 3:**
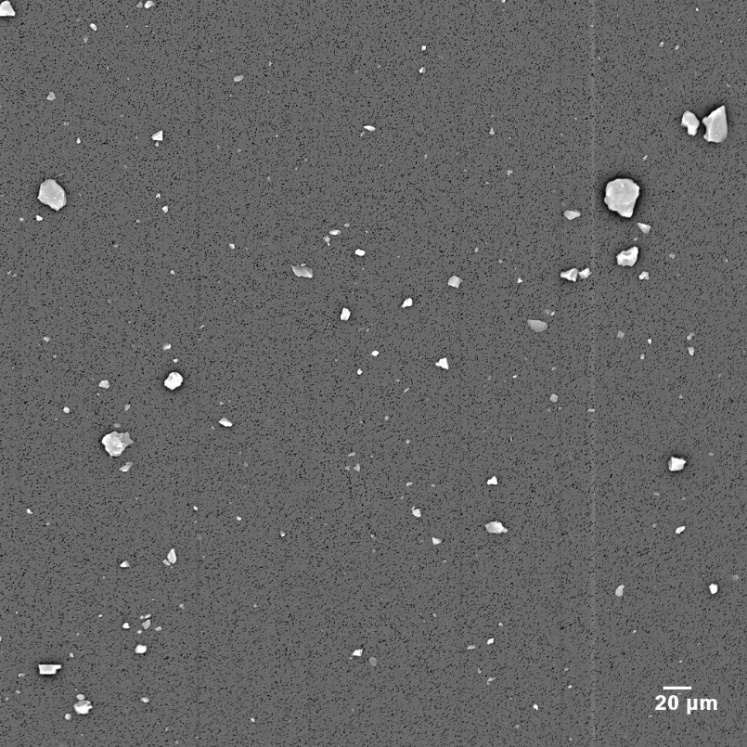
SEM micrograph of filtered candidate SRM 2806b with image field of 0.5 mm.

Figure 3 is a 0.5 mm by 0.5 mm magnified image of approximately 50 mL of candidate SRM 2806b. There are numerous Si-based particles, which represent the trace mineral dust added to make the standard.

**Fig. 4:**
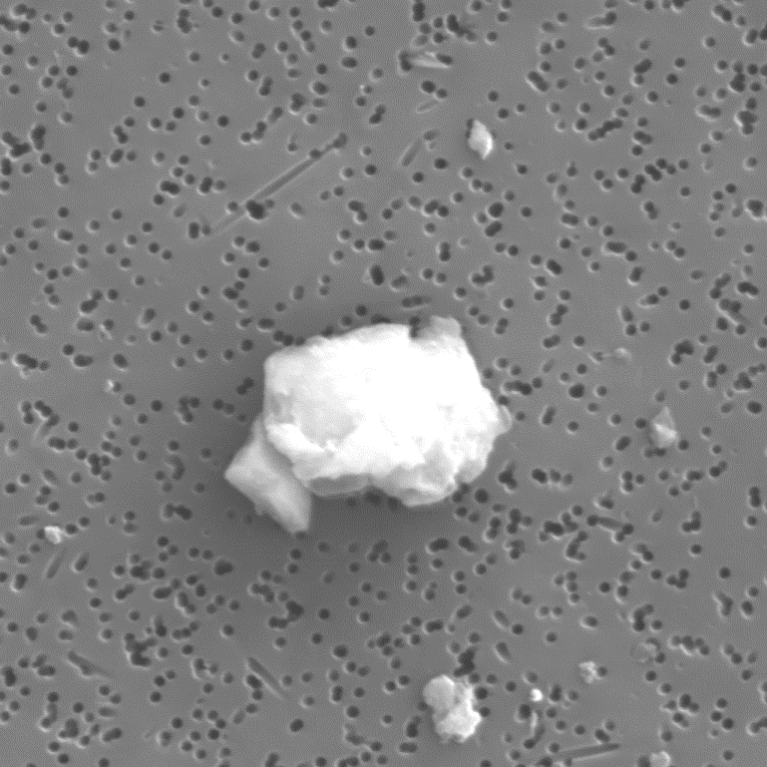
SEM micrograph of particles filtered from the refrigerated hydraulic oil for SRM 2806b. The image has a magnification of 5000×. The image is 25.6 μm on side, and the filter pore size is a nominal 0.4 μm.

The 5000× magnification of the SRM 2806b filtrate seen in Fig. 4 shows the mineral dust, but no contaminate particles. No contaminates other than the RM 8631 dust particles were found in any of the images obtained on the filters examined.

## 4 Statistical Testing of the Automatic Particle Counter Data (Five Steps)

A five-step statistical methodology was designed to quantify the quality of the candidate reference material, to aid the manufacturer and to provide firm evaluation criteria for NIST acceptance and continued work on a specific batch of material. The five steps are:

(1) distributional check: histograms,
(2) distributional check: normal probability plots,
(3) homogeneity and stability plots,
(4) homogeneity and stability statistics, and
(5) homogeneity and stability summary graphics.

In general, EDA (Exploratory Data Analysis) methods [6] for data processing are graphical procedures which prove to be informative and effective; this is due to the fact that the eye is intrinsically excellent at detecting anomalies (and structure) in a dataset. Our EDA method produces five pages of graphical output that are designed to display all the relevant information about a particular statistical aspect of the data on the same page. The graphical output is arranged with multiple plots/cells per page—one plot/cell for each diameter under investigation. The typical number of diameters per page is eight or sixteen. The data used to form the plots are cumulative particle concentrations; for example, the response for diameter 4 (say) is the total number of particles per milliliter with diameter size >4 μm. Because of the cumulative nature, the various diameter responses are not independent *per se*. For the five-step methodology, four out of the five steps are graphical; step 4 is quantitative. We briefly summarize the five steps here and then apply them to four separate examples (three from SRM 2806b production process in the year 2010 to 2011 and one from the SRM 2806d production process in 2018) to illustrate their diagnostic nature and their efficacy in accepting/rejecting a candidate SRM reference material. 

### 4.1 Distributional Check: Histograms


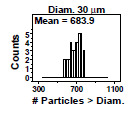
 This is a check for distributional shape and outliers. Under rather general circumstances, a well-behaved manufacturing process will have a histogram that is bell shaped and free of outliers. This first step produces a page of multiple histograms—one histogram for each particle diameter (typically 8 to 16 diameters). For a given diameter, the histogram shows the optical particle counter response (cumulative number particles per milliliter > the given diameter), which is ideally bell shaped and free of outliers. Non-bell-shaped histograms, skewed histograms, or outliers may be indicative of either (1) a material manufacturing process not in statistical control, or (2) the need for a change of units (*e.g.*, log transforms) in which to carry out all remaining analyses. For brevity, the mean particle size for a given plot is indicated at the top of the plot in an abbreviated manner; in this example, “Diam. 30” indicates a mean particle diameter of 30 μm.

### 4.2 Distributional Check: Normal Probability Plots


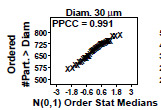
 This is a check for distributional normality. If the data are normally distributed, then certain subsequent statistical tests are more valid; non-normality is sometimes a flag for a process abnormality. This second step produces a page of eight or sixteen normal probability plots—one for each diameter under investigation. For a given diameter, the normal probability plot has the ordered response (cumulative number of particles per milliliter > the given diameter) on the vertical axis and theoretical normal-spaced values on the horizontal axis. Linearity in the plot indicates normality; non-linearity indicates non-normality. The associated normal probability plot correlation coefficient (PPCC) shown in each frame is a formal test of normality [7]. As before, “Diam. 30” indicates a mean particle diameter of 30 μm.

### 4.3 Homogeneity and Stability Plots


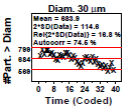
 This is a check for the homogeneity and stability of the material manufacturing process—a process in statistical control will be both homogeneous and stable. Homogeneity here means that the particle counts are nearly equivalent (as per analysis of variance [ANOVA] or Student’s *t*-test) between the first half of the manufacturing process and the second half. Stability means that the slope of the data over the entire manufacturing range is nearly flat. This third step produces a page of eight or sixteen scatter plots—one plot for each diameter. The vertical axis is the response (cumulative particle concentration); the horizontal axis is coded time (or ordered bottle). Low numbers indicate suspensions produced early in the manufacturing process, and high numbers indicate those produced later in the process. The plot is augmented with the mean value and with a band in which approximately 95% of the values should fall (if the process is in statistical control. The statistical quantities indicated in the plot are described below in Sec. 5.3.

### 4.4 Homogeneity and Stability Statistics


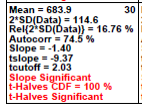
This is a quantitative analog for step 3, providing relevant formal test statistics to assess the homogeneity and stability of the material manufacturing process. This fourth step produces a page of eight or sixteen tabular cells—one cell for each diameter. The cell provides a list of test statistics that reflect the location and variability of the diameter’s response over the entire manufacturing range. The last three lines of the cell provide test statistic results for homogeneity and for stability. A well-behaved measurement process will pass these tests (and be colored as green). A process not in statistical control will fail these tests (and be colored as red). CDF indicates cumulative distribution function, and the statistical quantities indicated in the plot are described below in Sec. 5.4. 

### 4.5 Homogeneity and Stability Summary Graphics


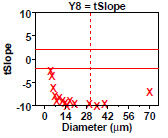
This is a graphical summary of all of the information presented in step 4. This fifth step has nine plots—one plot for each of the base statistics in step 4. The horizontal axis for each plot is the diameter (with typically eight or sixteen values). The vertical axis is the computed statistic (from step 4) for each diameter value. The most important plots are plots 8 and 9, which present the results of the homogeneity and stability statistics of step 4; the tests statistics are presented in green if the process "passed" and in red if the process "failed."

### 4.6 Statistical Testing Application

This five-step EDA methodology was applied to both early on-line data at the material developmental stage by the manufacturer and also later in bulk bottled samples at the final NIST SRM material acceptance stage. The combination of (1) focusing on key statistical properties of a process in statistical control, along with (2) the graphical nature of the approach enabled the material manufacturer and the reference material certification agency to immediately gain insight into problems with the data or the prospective sample.

The five-step procedure was designed and coded at the NIST and is embedded in code in the NIST-developed statistical software package (DATAPLOT). This package is portable and free to the public from https://www.nist.gov/itl/sed/dataplot [8]. The code (available on request) may be especially valuable to secondary suppliers of material to industrial customers (please contact james.filliben@nist.gov).

The following four sections illustrate the role that the five-step statistical procedure plays in assessing the quality of four different candidate material versions (at various stages in the manufacturing developmental process) of the NIST SRM 2806 reference material. The first three cases deal with 2806b (first sold in 2014), and the last case deals with 2806d (released in 2021):

Study 1: Rejecting an early stage (Dec. 2010) on-line SRM 2806b precandidate material;

Study 2: Accepting a later stage (July 2011) on-line SRM 2806b precandidate material;

Study 3: Accepting the final bottled SRM 2806b (July 2011) candidate material; and

Study 4: Accepting the final bottled SRM 2806d candidate material (July 2018).

The analysis will be presented, and interpreted, and accept/reject conclusions will be drawn.

## 5 Study 1: Rejecting an Early SRM 2806b On-Line Precandidate Material

The overall candidate material development process spanned many months. Iterative material production modifications were made frequently. The five-step statistical methodology was used continuously to monitor quality and improvement. This first example is from an early stage of material development.

In Dec. 2010, the manufacturer (IFTS) initiated the collection of on-line automatic optical particle data (cumulative particle concentration) for material made to specifications described in the *Specification for Manufacturing and Acceptance of New SRM 2806* created by an *ad hoc* ISO committee composed of Chinese, French, German, and U.S. representatives that included NIST (Appendix). The on-line data presented to NIST were the raw, unfiltered cumulative number of particles > specified diameter found in 50 mL of hydraulic fluid suspension for 16 particle diameters. The mean cumulative particle concentration values were consequently large.

The five-step statistical methodology is as follows:

### 5.1 Distributional Check: Histograms (Early 2806b)

**Fig. 5:**
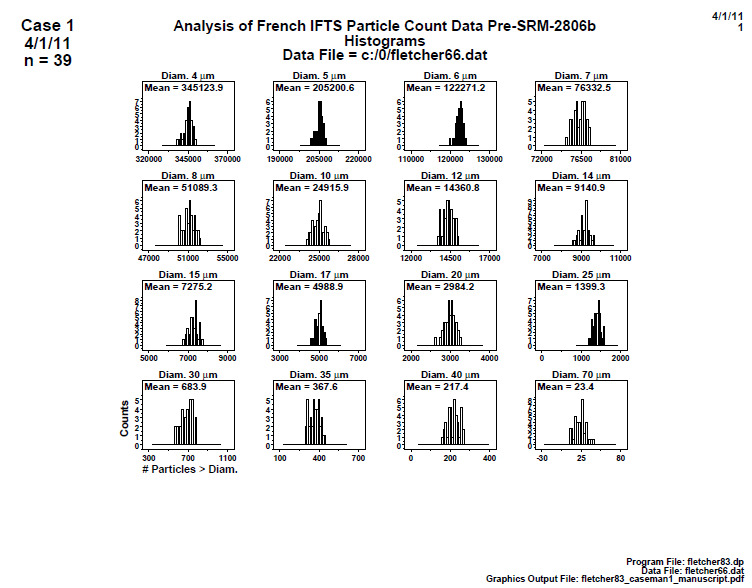
Results for SRM 2806b early on-line data step 1: distributional check: histogram. Axis labels for all plots are the same as those given on the lower-left plot.

There are 16 plots in Fig. 5, one for each particle diameter. Each plot contains 39 individual readings taken at sequential times during the 2 h material production process. The vertical axis is the histogram frequency/counts; the horizontal axis is the particle counter response, which is the cumulative number of particles > specified diameter. The mean value is given in the upper left of each plot. The ideal shape would be a normal distribution in a symmetric bell-shaped histogram.

Conclusion 1: The mean values decline (as they must) for increasing diameter. The first few (smaller) diameters show some skewness, which may be consistent with the underlying Poisson nature of particle count data. No outliers in the particle count data are evident.

### 5.2 Distributional Check: Normal Probability Plots (Early 2806b)

**Fig. 6:**
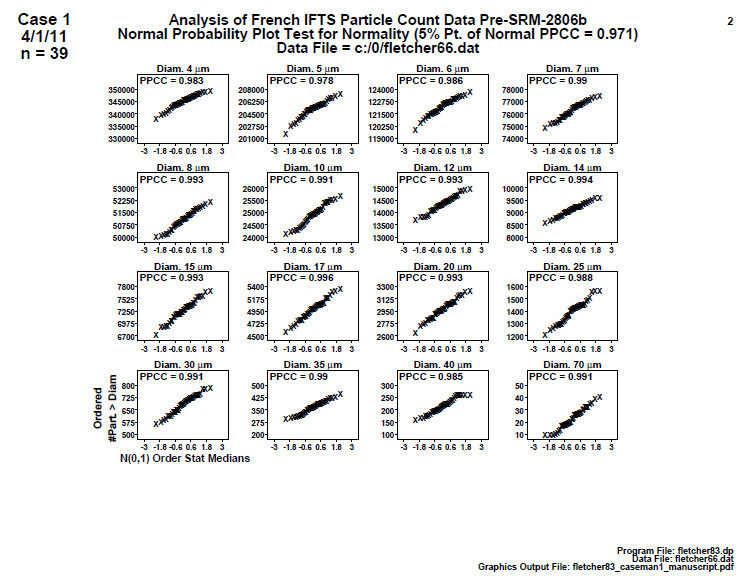
Results for SRM 2806b early on-line data step 2: distributional check: normal probability plots. Axis labels for all plots are the same as those given on the lower-left plot.

In Fig. 6, the same data are further analyzed for normality. The same number of plots are produced that correspond to the histogram plots in Fig. 5. The vertical axis is the sorted response (cumulative number of particles > specified particle diameter), and the horizontal axis contains the theoretical ordered values for a normal N(0,1) distribution. Linearity implies normality; data closer to the line indicate more normal data.

If the vertical axis response data (the cumulative number of particles > the diameter) happen to be normal, then this plot will be a plot of two sorted normal sequences and hence will be linear. The normal probability plot correlation coefficient (PPCC) appearing above each plot formalizes that linearity (and hence normality) via a test statistic. A perfect (= linear) normal probability plot will have a normal PPCC value of 1.000. Near-perfect (= near-linear) normal probability plots will have a normal PPCC close to 1.000. Poor (= non-linear) normal probability plots will have a normal PPCC much smaller than 1.000. Note that for *n* = 39 points, the 5% significance cutoff value for the normal PPCC statistic is 0.970 [7]. PPCC values in the interval (0.970, 1.000) will indicate normality, while PPCC values ≤ 0.970 will be statistically significantly non-normal.

Conclusion 2: The plots are generally linear (normal), with the exception of the first few (smaller) diameters, which exhibit some minor curvature. The three diameters closest (but not statistically significant) to the 0.970 cutoff value are diameter 4 (PPCC = 0.983), diameter 5 (PPCC = 0.978), and diameter 6 (PPCC = 0.986). In terms of outliers, no outliers were evident.

### 5.3 Homogeneity and Stability Plots (Early 2806b)

**Fig. 7:**
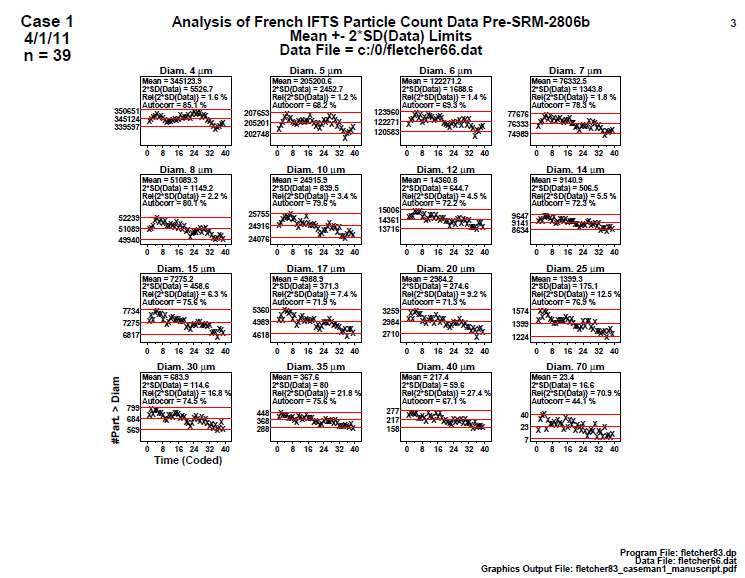
Results for SRM 2806b early on-line data step 3: homogeneity and stability plots. Axis labels for all plots are the same as those given on the lower-left plot. The horizontal axis represents the 39 measurements which are distributed over the approximate 2 h sampling time.

Step 3 utilized the same 39 data sequential points collected during material production (Fig. 7) with the vertical axis showing the cumulative number of particles > diameter and the horizontal axis showing the coded ordered number (1 to 39) over the material production time; hence, small values were collected early in the on-line measurement process, and larger values were measured later in the process. The mean is shown in upper left and corresponds to the center line in the plot, with the outer lines positioned at approximately the 95% data coverage (assuming normality). In the legend, “Autocorr” is the autocorrelation coefficient test, a statistic that evaluates any hidden systematic variations or trends. The autocorrelation ranges from –100% to 100%. If random (the ideal), Autocorr will be near 0.

These statistically augmented scatter plots are excellent diagnostic tools for detecting lack of randomness, drift, and inhomogeneity in the production line data. They represent one of the most significant diagnostic tools in the five-step analysis process.

Conclusion 3: These plots are effectively a set of time-series plots from the beginning to end of production. It is clear why this on-line data set was defective. For each and all 16 diameters, the data have a (severe and significant) negative slope. The production process is in fact yielding material in which the particle count data are drifting down over time. Also, visually for any given diameter, the mean of the data for the second half is not equal to (and is less than) the mean of the data for the first half. This plot thus indicates that the manufacturing process is not (yet) in statistical control with respect to homogeneity (first half versus second half equivalence) nor with respect to stability (no drifting).

Four statistics are given in the upper-left legend:

(1) the mean particle concentration,
(2) twice the standard deviation (SD) of the data (approximate 95% variation limits),
(3) relative variation limits [2×SD(data)/Mean(data) as a percentage], and
(4) the autocorrelation coefficient, Corr (Y(*i*),Y(*i*+1)) [−100%,+100%].

For a process in statistical control, the autocorrelation will be near zero. For this process (and for all of the diameters), this process has excessively large autocorrelations, which indicate that the 39 data points are NOT behaving like 39 random drawings from some fixed material particle size population – in effect there appear to be fewer than 39 independent “snapshots” of the process.

The serious practical effect of this condition is that the usual uncertainty formula for the standard deviation in the mean (the standard error):

SD(mean) = SD(data)/sqrt(*n*) (1)

is no longer valid because we do not have the full contingent *n* = 39 independent drawings from a population; in fact, the data shows a clear sequence (drift). Hence, the computed SD(mean) (which is needed for the uncertainty statement for the reference value on the final SRM certificate) would in fact be optimistically smaller than the true SD(mean) that should be computed. This process does in fact have some smaller number of independent observations than *n* = 39, and hence the true SD(mean) is larger, as computed as

SD(mean) = SD(data)/sqrt(n*) (2)

where n* is some unknown value < 39.

The dominant conclusion from these plots in step 3 is that the process is drifting from the beginning to the end of the material manufacturing process. Note that an inflated autocorrelation coefficient is quite typical for a process with significant drift. This step 3 analysis thus identified this early stage material manufacturing process output as defective, and because the process is not in complete statistical control, as indicated by the significant downward trend in particle concentration over time, the material was deemed unacceptable as a reference material.

### 5.4 Homogeneity and Stability Statistics (Early 2806b)

**Fig. 8:**
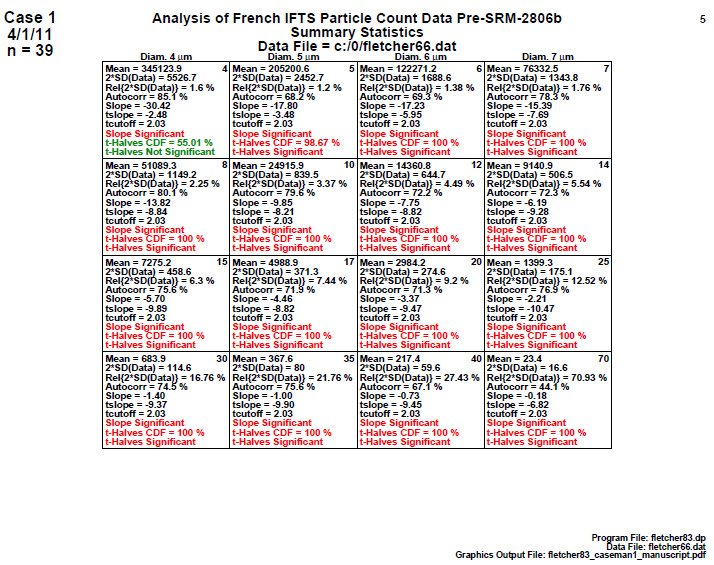
Results for SRM 2806b early on-line data: Step 4: Homogeneity and Stability statistics.

Step 4 in Fig. 8 quantitatively summarizes the graphical information presented in the plots of step 3. Each cell corresponds to a particle diameter with the same 39 members. There are 10 statistics within each cell representing a collection of location, variation, slope, and homogeneity statistics. The most important statistics are the last three, which show the results of a slope (stability) test and a first-half versus second-half ANOVA (homogeneity) test and its significance. If the material passes the statistical test, the result is colored as green; the result is red if it fails.

Conclusion 4: For all 16 diameters, the final three lines show that the slope is red (= statistically significant), which indicates the process is drifting (high-to-low) over the 2 h duration of the material production process. Further, in 15 out of the 16 diameters, the red ANOVA ("t-Halves") also indicates that the process is not homogeneous when considering the first half of the data versus the second half. Step 4 thus formally tested/affirmed what step 3 graphically implied, namely, that the manufacturing process is not in statistical control with respect to homogeneity (second half versus first half) nor with respect to stability (no drift). Clearly for this early-stage production run, there was a material production mixing issue that would be corrected in later stages.

Details: For a given diameter (Fig. 8), the 10 statistics presented within each cell are as follows:

1. the mean cumulative particle concentration (Mean) [particles/mL],
2. twice the standard deviation of the data [2×SD(data), approximate 95% data variation limits] [particles/mL],
3. relative variation limits [2×SD(data)/Mean(data)] as a percentage [%],
4. the autocorrelation coefficient (Autocorr), Corr (Y(i),Y(i+1)) [-100%,+100%],
5. the slope (Slope) [particles/volume/time],
6. the *t*-value for the (tslope = slope/SD(slope),
7. the 95% cutoff value (for *n* = 39 observations) for the *t*-value for the slope,
8. a statement as to whether the slope is significant,
9. the cumulative distribution value for ANOVA statistic (t-Halves CDF, significant if ≥ 95%), and
10. a statement as to whether the ANOVA statistic (t-Halves) is significant.

Again, the dominant conclusion from the presented statistics of this step 4 is that the material manufacturing process is drifting down from beginning to end. Bottles produced in the second half of the process have (on average) fewer numbers of particles > a given diameter than bottles produced earlier in the process.

Steps 3 and 4 thus identified and concluded that this early-stage material production process is not in statistical control and is drifting, and that the output from this early-stage production process is not yet ready to be acceptable as a candidate reference material.

### 5.5 Homogeneity and Stability Summary Graphics (Early 2806b)

**Fig. 9:**
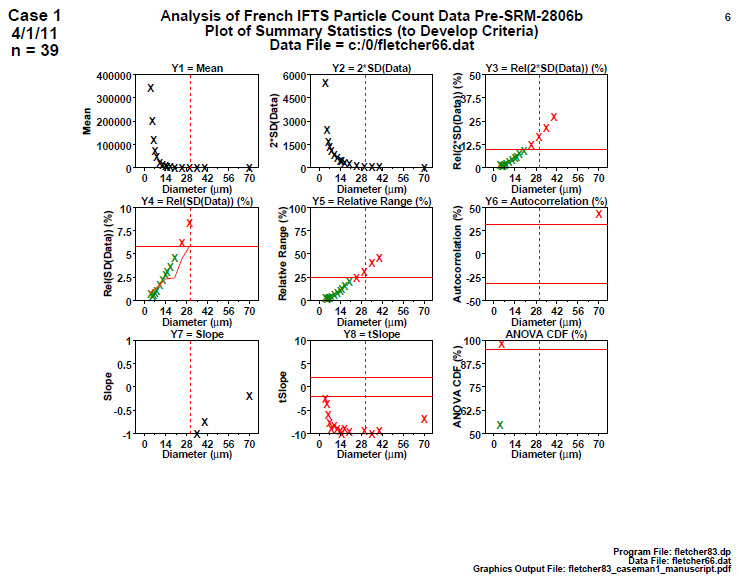
Results from SRM 2806b early on-line data for 2806b step 5: homogeneity and stability summary graphics. This graphic is a summary of the step 4 information.

Step 5 summarizes the results for the early on-line 2806b material with nine plots (Fig. 9), one for each of the nine computed statistics in step 4. All 16 diameters are plotted with vertical axis having the computed statistic and the horizontal axis showing the diameter.

The dotted red vertical bar in the plots is at diameter = 30 μm because the scope of our conclusions for SRM 2806 is in the diameter range (4 to 30) μm. Values beyond 30 μm are not certified.

Conclusion 5: Of lesser interest (from plots 1 to 7), it is seen that larger diameters have smaller means, smaller 2×SD(Data), and larger relative 2×SD(Data), larger relative ranges [100×(max−min)/mean], and smaller slopes. Plot 6 has most of its points off scale to the high side showing that nearly all autocorrelations are too large and statistically significant. Plot 7 has most of its points off the plot to the low side showing that all slopes are negative. Of major interest (from the last two plots), plot 8 indicates that (for all 16 diameters) the computed slopes are statistically significantly negative (red), and so the process is not stable; plot 9 indicates that (for 15 out of 16 diameters) the computed ANOVA *F* statistics are statistically significant (red), and so the process is not homogeneous.

From a quality metric point of view, all the values found outside (red) the allowable bands in plots 8 and 9 may be summed up to yield a quality score, where large is poor, and zero is perfect. The quality score for this early-stage data is 16 (from plot 8) + 15 (from plot 9) + 6 from other frames, thus yielding a total score of 37. This large value is poor and indicates a drifting, inhomogeneous process not in statistical control.

This early-stage candidate material had a trend in the cumulative particle concentration as a function of time when the bottle of test material was produced during the approximate 2 h fill process. The particle concentration decreased with production time. The trend is illustrated and clearly visible in step 3 and reaffirmed in step 4. It is possible that bias was due to the mixing process, as evidenced by the on-line optical particle data, which indicated there was a systematic change in concentration during the 2 h bottle-filling process. The falling slope and the statistical disagreement between the first half and last half of the production cycle made this potential candidate material unacceptable. As a result, the manufacturer continued to iteratively modify and improve their production process over the next few months, with the objective to produce a superior product.

## 6 Study 2: Accepting a Later SRM 2806b On-line Precandidate Material

As a result of the early-stage five-step analysis results, repeated adjustments were made by the manufacturer to optimize the process, and *in situ* on-line quality monitoring via execution of the five-step process provided immediate evaluation of the effectiveness of the process iterations. This rapid on-site/on-line feedback was essential in yielding an optimized candidate reference material in a relative short amount of time (a few months).

This second case study presents results of the five-step analysis as applied to a later stage (about iteration 10) of the material-improvement process. The first case study had *n* = 39 measurements under analysis. The number of measurements for this second case study is larger: *n* = 68. The measurement identifiers (IDs) (1 to 68) reflect their (coded) time order in the larger production process.

The following plots will show that this later-stage material is far superior to the original "blend" and was much closer to being an acceptable candidate reference material. The same five steps were applied:

(1) distributional check: histograms,
(2) distributional check: normal probability plots,
(3) homogeneity and stability plots,
(4) homogeneity and stability statistics, and
(5) homogeneity and stability summary graphics.

### 6.1 Distributional Check: Histograms (Later 2806b)

**Fig. 10:**
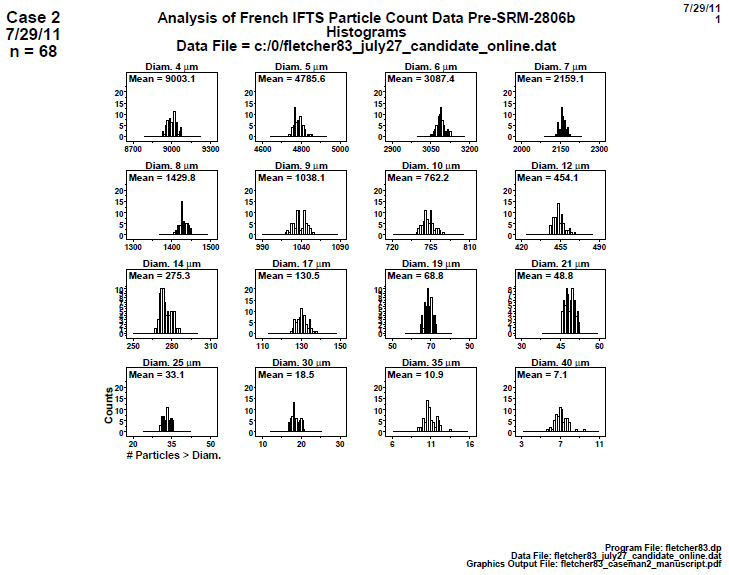
Results of SRM 2806b later on-line data step 1: distributional check: histogram. Axis labels for all plots are the same as those given on the lower-left plot.

Figure 10 presents the 16 diameters and 68 data points taken from an on-line sample of prospective SRM 2806b. The histogram counts are shown on the vertical axis, and the 68 optical particle counter responses (the cumulative number of particles per milliliter > the given diameter) are shown on the horizontal axis. The 16 histograms are shown, though only 14 were used (since only diameters ≤ 30 μm are needed for the SRM).

Conclusion 1: The histograms are generally symmetric, and no skewness appears (as was the case with the case 1 smaller diameters). For most diameters, no outliers appear; for the two largest diameters (35 and 40) μm, a few high-side possible outliers exist. By default, this would generally warrant a follow-up Grubbs test for normal outliers (though diameters 35 μm and 40 μm are not determined for the SRM).

### 6.2 Distributional Check: Normal Probability Plots (Later 2806b)

**Fig. 11:**
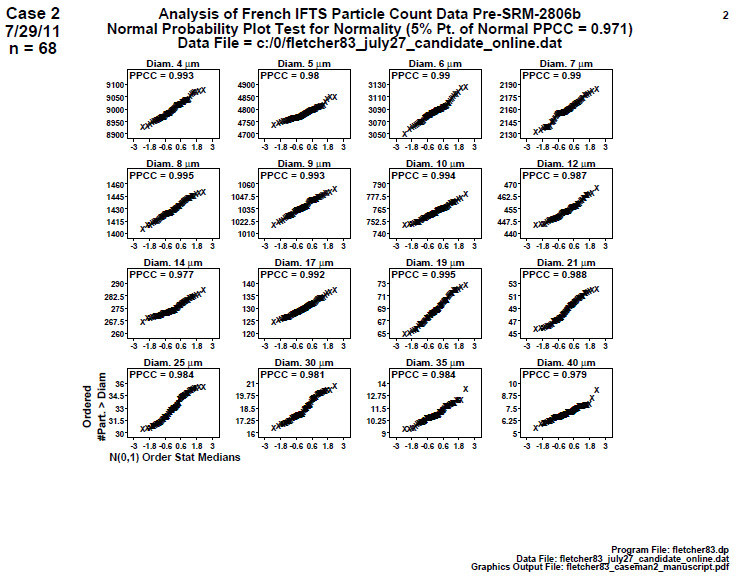
Results of SRM 2806b later on-line data step 2: distributional check: normal probability plots. Axis labels for all plots are the same as those given on the lower-left plot.

In step 2, a distributional check of normal probability via probability plots (Fig. 11) would ideally show a set of lines for normally distributed data. The vertical axis is the cumulative number of particles per milliliter > specified diameter. The horizontal axis is the 68 sorted theoretical values from a normal N(0,1) distribution.

Note that for *n* = 68 points, the 5% significance cutoff value for the normal PPCC statistic is 0.982. PPCC values in the interval (0.982,1.000] will indicate normality, while PPCC values ≤ 0.982 will be statistically significantly non-normal.

Conclusion 2: All plots are nearly linear. Since the 5% significance cutoff for the normal PPCC statistic is 0.982, any plots with PPCC values ≤ 0.982 will fail as normal. The normal PPCC test passes for most (13 out of 16) diameters, and for these plots, the normality assumption is "accepted." The normal PPCC test fails for three diameters: diameter 14 μm (PPCC = 0.977), diameter 40 μm (PPCC = 0.979), and diameter 30 μm (PPCC = 0.981), and for these plots, the normality assumption is rejected.

Outlier-wise, the plotted points look very much in line for most (14) of the 16 diameters. For diameter = 35 μm, the largest observation looks suspicious, as do the two largest values for diameter = 40 μm. Subsequent Grubbs tests indicated that these potential outliers were not statistically significant (though Grubbs test assumes normality, which does not exist here).

### 6.3 Homogeneity and Stability Plots (Later 2806b)

**Fig. 12:**
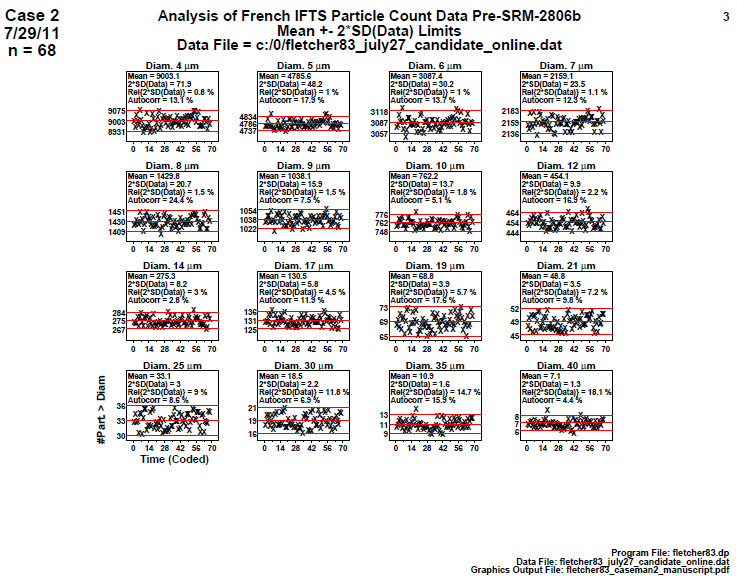
Results of SRM 2806b later on-line data step 3: Homogeneity and Stability Plots. Axes labels for all plots are the same as those given on the lower left plot. The horizontal axis is the 68 measurements which are spaced over the approximate 2 h sampling time.

For the second on-line data set, a time-series set of scatter plots was generated starting with early samples on the left and ending with final values on the right, as shown in Fig. 12. For the on-line data of 68 points, the values do not correspond exactly to the 40 bottles but are nearly equivalent because the duration of the on-line analysis is known. Data were broken into particle size segments, with the diameter of the particles under examination shown in the upper-right corner. Both the mean value timeline and the approximate 95% data bands are shown. As before, these bands were computed as Mean ± 2×SD(Data). The information in these plots visually indicates the percentage of the data points falling between these two bands (if normal, then approximately 95%). Compared to the first (early-stage) on-line data set given in study 1, this second (later-stage) on-line data set proves to be much better, passing the statistical tests with no difficulty.

There are 16 plots (one for each of the 16 diameters) with 68 data points. The vertical axis is the optical particle counter response, in terms of cumulative number of particles per milliliter > the given diameter, and the horizontal axis is the coded sample ID (ordered over material production time). Autocorr is the autocorrelation test statistic.

Conclusion 3: Unlike study 1 (early-stage data), the study 2 on-line data have a near-zero slope for all 16 diameters. This later-stage material manufacturing process appears to be quite stable (and predictable) over time. Visually, for any given diameter, the mean of the data for the plot’s second half is approximately the same as the mean of the data for the first half. This plot thus suggests that the manufacturing process is (visually) in statistical control with respect to homogeneity (second half versus first half) and also with respect to stability (near-zero slope and no apparent drifting). These graphical results of step 3 indicate that the efforts to improve the material production process in going from (study 1) early stage to (study 2) later stage are appearing to be successful.

### 6.4 Homogeneity and Stability Statistics (Later 2806b)

**Fig. 13:**
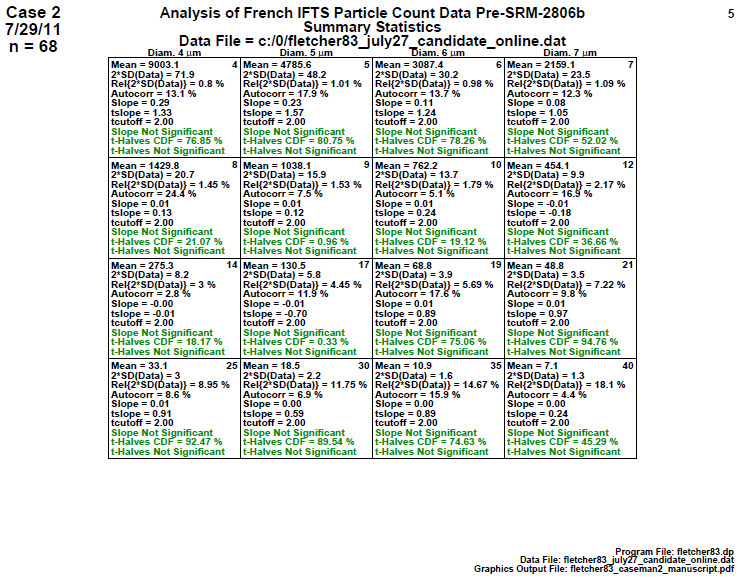
Results of SRM 2806b later on-line data step 4: homogeneity and stability statistics.

This step 4 exhibit (Fig. 13) quantitatively summarizes the graphical information presented in the plots of step 3. The number of statistics within each cell is 10, representing a collection of location, variation, slope, and homogeneity statistics. The most important statistics are the last three; these final three lines within each cell show the results of a slope (stability) test and a first half versus second half ANOVA (homogeneity) test and its significance. Figure 13 is dominated by green which indicates that all the statistical tests were passed.

Conclusion 4: For all 16 diameters, the slope is near zero (statistically insignificant), which indicates the process is stable over the 2 h lifetime of the material manufacturing process. Also, for all 16 diameters, the green ANOVA indicates that the two time-halves of the process are statistically equivalent (homogeneous). Step 4 thus formally affirms that considerable process improvement has been achieved in going from the early-stage results (study 1) to the later-stage results (study 2). This later-stage manufacturing process appears to be very much in statistical control both with respect to homogeneity (second half versus first half) and with respect to stability (near-zero slope).

The material from this later-stage process would serve as a strong potential candidate for being used as the SRM 2806b candidate material.

### 6.5 Homogeneity and Stability Summary Graphics (Later 2806b)

**Fig. 14:**
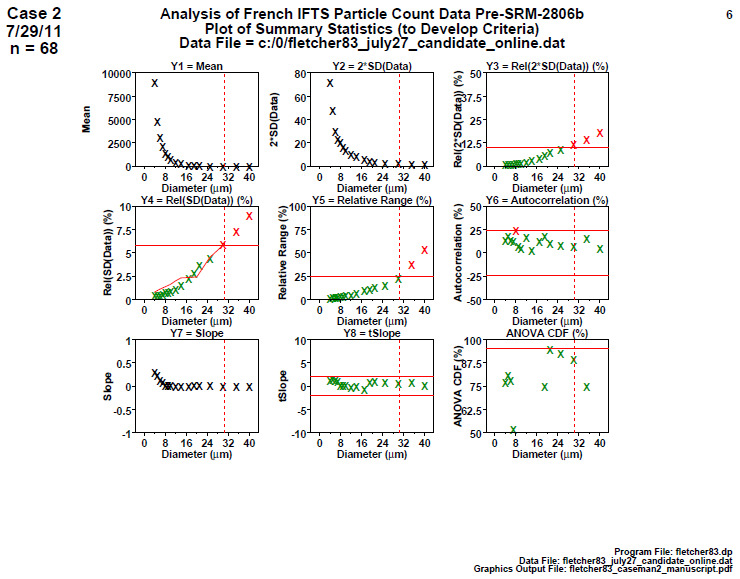
Results of SRM 2806b later step 5: homogeneity and stability summary graphics.

Figure 14 is a summary of the step 4 information. There are nine plots, one plot for each of the nine computed statistics in step 4: plot 1=Mean, plot 2=2×SD(Data), plot 3=relative[2×SD(Data)], *etc*. There are 16 diameters (ranging from 4 μm to 40 μm) presented on the horizontal axis, and the computed statistic appears on the vertical axis. There is a (dotted red line) cutoff at diameter 30 μm, which is the diameter upper limit to be used for certification for SRM 2806b.

Conclusion 5: As before, higher diameters have smaller means, smaller 2×SD(Data), larger relative 2×SD(Data) and larger relative ranges. Plot 6 indicates well behaved statistically insignificant autocorrelations. Plot 7 indicates near zero slopes. The last two plots are the most important because they test whether the slopes are statistically insignificant (stability) and whether the two halves of the data are statistical equivalent (homogeneity). Contrary to the study 1 early-stage data, this study 2, later-stage data are excellent: For all diameters, the slope is statistically flat (green), indicating a stable process, and the ANOVA is statistically equivalent (green), indicating a homogeneous process.

From a quality metric point of view, the sum of all the values found outside (red) the allowable bands in plots 8 and 9 yields a score of 0, and plots 3, 4, and 6 have a total of 3, thus yielding a total score of 3. This small value is excellent and indicates a stable, homogeneous process in statistical control.

#### 6.5.1 Overall Five-Step Analysis Conclusions (Later SRM 2806b)

This later-stage candidate material was excellent in that the cumulative particle concentration was invariant and did not depend on when the bottle of test material was produced during the approximate 2 h fill process time. The stability and homogeneity of this later-stage material indicate that the process product (material) had improved to the point of approaching acceptability for use as the candidate SRM 2806b material. The quality of this later-stage material was a tribute to the (1) effort put forth by the manufacturer to iteratively improve the product over 7 months, and (2) the importance of this five-step methodology being used on-line, which gave the manufacturer a tool with which the data could be analyzed immediately to provide rapid feedback, to identify problems, and to ascertain the success of a production modification.

## 7 Study 3: Acceptance of the Final SRM 2806b Candidate Material

It is one thing to obtain acceptable mixing in the circulating test stand with an on-line particle counter, and yet another to obtain good bottle-to-bottle homogeneity in the bottled material. NIST acceptance is based not on the on-line results, but only on the finished bottled product. The bottled material by its very nature will have higher variability. The final candidate material for SRM 2806b was the result of 7 months of process improvement by IFTS, of which case study 1 above was a (rejected) early-stage example and case study 2 above was a (near-acceptable) later-stage example. The final candidate material for SRM 2806b was delivered by IFTS to NIST in July of 2011.

As alluded to in study 2, repeated adjustments (instrumental/procedural/environmental) were made by IFTS over the prior 7 months to optimize the manufacturing process. Each adjustment was evaluated (locally by IFTS and remotely by NIST) via the five-step battery of statistical tests. From step 5 of the procedure, a metric was developed to quantify the quality of the resulting material product. The quality score provides the number of failed (step 5) tests (specifically, in plots 1, 2, 5, 6, 7, 8, and 9 of step 5) in a material, and so, ideally, the perfect quality score is zero.

Table 1 shows the quality scores from a selected subset of the iterative runs made by IFTS over the 4 months spent improving the manufacturing process. The earliest manufactured material had a score of 37 failures, which decreased monthly to only three failures for the final material on-line data (and 0 for NIST’s follow-up tests on a 40-bottle subset). This is the material that NIST ultimately accepted for further certification for SRM 2806b.

**Table 1:** Results of statistical testing of manufactured material that show the progress toward refining the manufacturing process. A quality score of 44 means that there were 44 data points outside the step 5 acceptable boundaries (and hence the material was unacceptable as a standard due to failures in both stability and homogeneity). NIST was using a criteria score of <4 for acceptance. After multiple iterations, a score of zero was achieved by IFTS and confirmed by NIST analysis.

**Manufactured (Date)**	**Quality Score (Zero = Perfect)**	**Study Number**	**On-Line or Bottled**
M1 (12-07-2010)	37	1	On-line
M1 (5-30-2011)	12		On-line
M1 (6-22-2011)	10		On-line
M2 (5-11-2011)	9		Bottled
M2 (5-11-2011)	6		Bottled
M1 (final) (7-26-2011)	3	2	On-line
M1 (final) (7-27-2011)	0	3	Bottled
NIST	0		Bottled

The previous two case studies applied to on-line data, which are important for diagnostic determinations. However, the fully bottled reference material is the most important because this is the finished product that will be used by the relevant industries. The five-step analysis as applied to this third case study for SRM 2806b (bottled material) is shown below.

The number of bottles for this third study is *n* = 40. Like usual, the bottle IDs (1 to 40) reflect their order in the larger production process with1 being near the beginning and 40 being near the end.

### 7.1 Distributional Check: Histograms (Final 2806b)

**Fig. 15:**
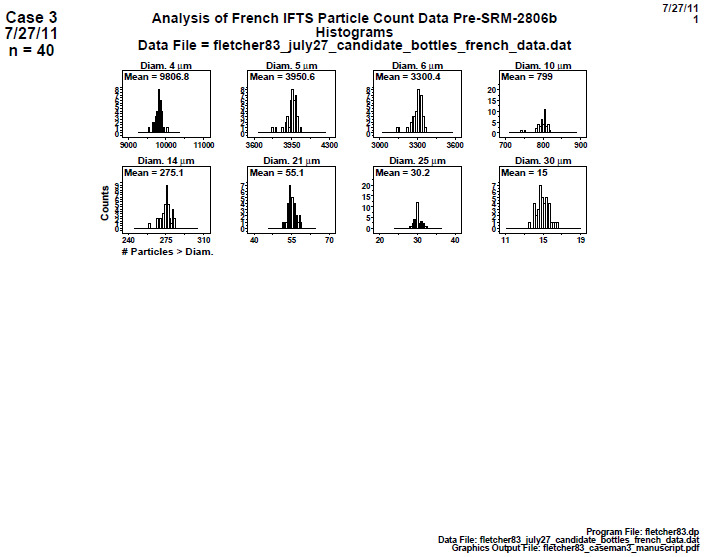
Results of SRM 2806b final material step 1: distributional check: histogram. Axis labels for all plots are the same as those given on the lower- left plot.

Figure 15 shows eight plots, one for each particle diameter and for the 40 bottle analyses obtained for each diameter. The vertical axis presents the histogram count or frequency, and the horizontal is the cumulative particle concentration > specified diameter.

Conclusion 1: The histograms for the larger (21, 25, 30) diameters are symmetric. The histograms for the smaller (4, 5, 6, 10, 14) are skewed to the left. There may be outliers at diameters 5, 6, 10, and 14.

### 7.2 Distributional Check: Normal Probability Plots (Final 2806b)

**Fig. 16:**
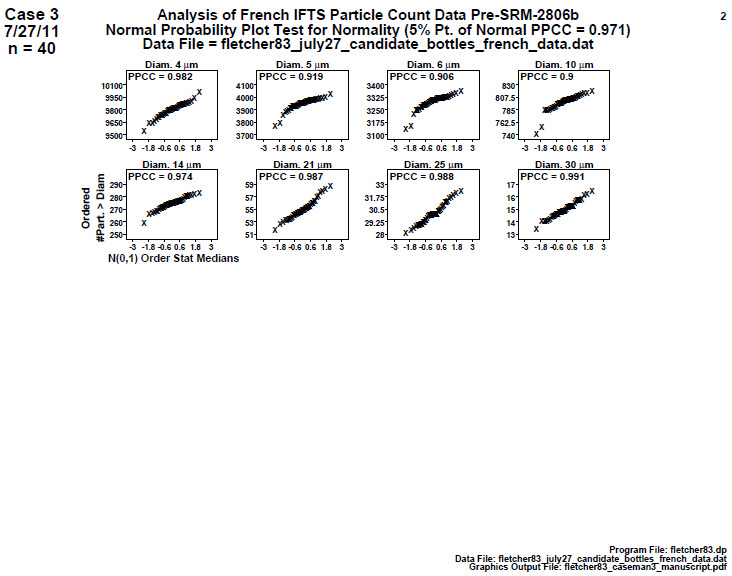
Results of SRM 2806b final material: Step 2: Distributional check: normal probability plots. Axis labels for all plots are the same as those given on the lower-left plot.

The normal probability plots (Fig. 16) provide the diagnostic tool with which to assess the degree of normality of the data, with the vertical axis presenting the cumulative particle concentration > specified diameter and the horizontal axis giving the sorted theoretical values from a normal N(0,1) distribution.

Note that for *n* = 40 points, the 5% significance cutoff value for the normal PPCC statistic is 0.972, and the 1% significance cutoff value is 0.958 [7]. Normal PPCC values > 0.972 indicate normality; values ≤ 0.972 indicate non-normality; values ≤ 0.958 strongly indicate non-normality.

Conclusion 2: Diameter 4 and the largest (14, 21, 25, 30) diameters all test out as normal. Diameters 5, 6, and 10 are problematic because they all fail normality, not only at the 5% level, but also at the 1% level. If the two outliers are removed, then diameters 5 and 6 will still fail normality, but diameter 10 will pass. Note that normality is ideal, and failing normality, though not fatal for an SRM, is less than ideal. The particle distribution is intrinsic to the material. In an SRM context, homoscedasticity and stability are more important than normality. On the other hand, if non-normal, it may at times reflect the existence of an outlier, which is to be noted and then dealt with in three ways:

(1) testing and removing the outlier correctly.
(2) accepting the fact that the outlier will invariably inflate the uncertainty of the final SRM certified value; and
(3) choosing wisely the statistic with which to estimate the final SRM certified value; for example, the mean will be affected by the outlier, while the median will be much more outlier-resistant (and hence in general would be preferred).

#### 7.3 Homogeneity and Stability Plots (Final 2806b)

**Fig. 17:**
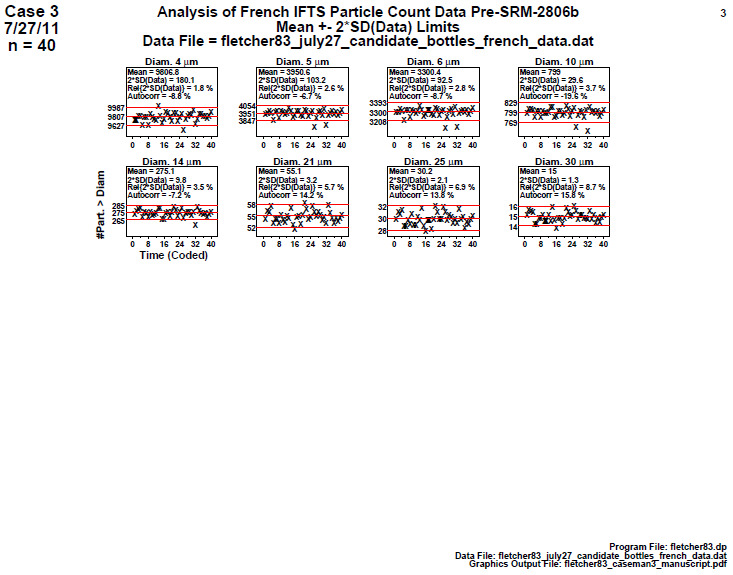
Results of SRM 2806b final material step 3: homogeneity and stability plots. Axis labels for all plots are the same as those given on the lower-left plot. The horizontal axis represents the 40 measurements (40 bottles) which are distributed over the approximate 2 h sampling time.

The scatter plots in Fig. 17 show lack of a trend in the data from start to end of the bottling process. The vertical axis is cumulative particle concentration > specified diameter, and the horizontal axis is the 40-bottle representation.

Conclusion 3: The SRM 2806b bottled data have near-zero slope for all eight diameters. This final-stage material manufacturing process thus appears to be quite stable (and predictable) over time. Visually, for any given diameter, the mean of the data for the plot’s second half is approximately the same as the mean of the data for the first half. This plot thus indicates that the manufacturing process is (visually) in statistical control with respect to homogeneity (second half versus first half) and with respect to stability (near-zero slope and no apparent drifting).

With respect to outliers, the two potential outliers in step 2 for diameters 5, 6, and 10 are showing up (as they must) as outside (below) the (95%) coverage limits for the step 3 plots of diameters 5, 6, and 10. Note that the values do look suspicious, but also note that these are 95% coverage limits, and so we would expect 1 in 20 to be outside the limits under perfect conditions, and so the 2 in 40 that we observe is not strikingly unusual. As mentioned before, the safer way to deal with suspicious data (outliers) is to estimate the final SRM certified value via the outlier-resistant median (as opposed to the outlier-sensitive mean).

### 7.4 Homogeneity and Stability Statistics (Final 2806b)

**Fig. 18:**
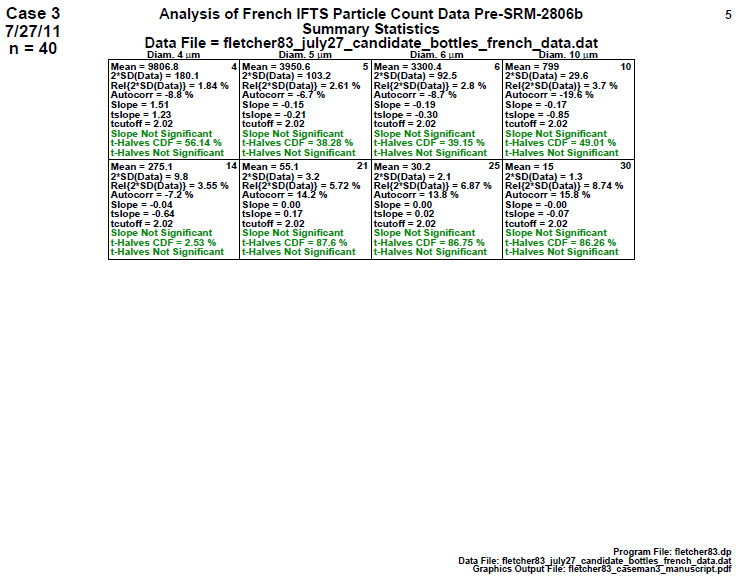
Results of SRM 2806b final material step 4: homogeneity and stability statistics.

This Step 4 quantitatively summarizes the graphical information presented in the plots of step 3. The most important statistics are the final three lines within each cell, which show the results of a slope (stability) test and a first half versus second half ANOVA (homogeneity) test and its significance.

Conclusion 4: For all eight diameters, the slope is near zero (statistically insignificant), which indicates the process is stable over the 2 h lifetime of the material manufacturing process. Also, for all eight diameters, the ANOVA indicates that the two time halves of the process are statistically equivalent (homogeneous). Step 4 thus formally affirms that the material from this final-stage process would well serve as a final candidate material for SRM 2806b.

### 7.5 Homogeneity and Stability Summary Graphics (Final 2806b)

**Fig. 19:**
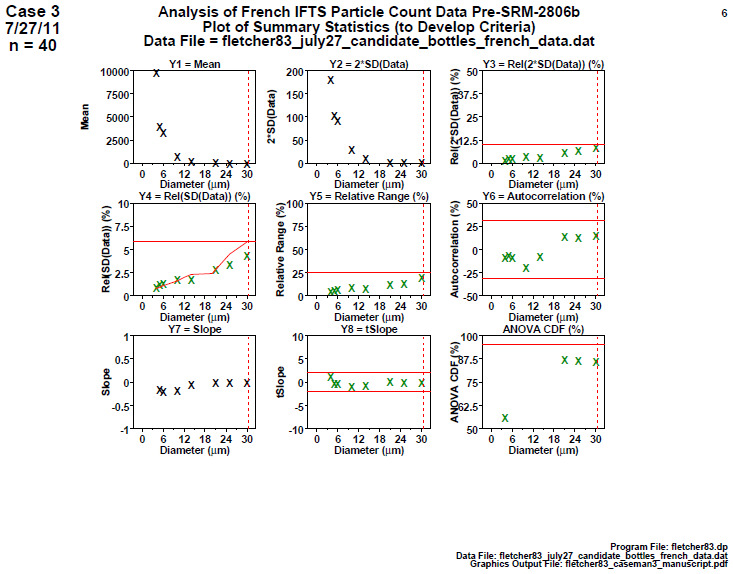
Results of SRM 2806b final material step 5: homogeneity and stability summary graphics.

Figure 19 is a summary of the step 4 information. The vertical axis is the computed statistic, and the horizontal axis presents the 30 particle diameters specified for SRM 2806b.

Conclusion 5: As before, higher diameters have smaller means, smaller 2×SD(Data), larger relative 2×SD(Data), larger relative ranges, and smaller slopes. The last two plots are the most important because they test whether the slope is statistically insignificant (stability) and whether the two halves of the data are statistically equivalent (homogeneity). Contrary to the study 1 early-stage data, and along with the study 2 later-stage data, these study 3 final-stage data are excellent: For all diameters, the slope is statistically flat (green), indicating a stable process, and the ANOVA is statistically equivalent (green), indicating a homogeneous process.

From a quality metric point of view, the sum of all the values found outside (red) the allowable bands in plots 8 and 9 yields a score of 0 (from plot 8) + 0 (from plot 9), thus yielding a total score of 0. This small value is excellent and indicates a stable, homogeneous process in statistical control.

#### 7.5.1 Overall Five-Step Analysis Conclusions (Final 2806b)

This final-stage candidate material was excellent in that the cumulative particle concentration was invariant and did not depend on when the bottle of test material was produced during the approximate 2 h fill process time. The stability and homogeneity of this later-stage material indicate that the process product (material) had improved to the point of acceptability for use as the candidate SRM 2806b material. As noted earlier, the quality of this final-stage material was due to (1) hard work by IFTS, and (2) the existence and on-line use of the five-step statistical quality assessment procedure. The net result was a delivered set of 400 bottles from IFTS that constitute the NIST SRM 2806b reference material.

A comparative analysis of the IFTS and NIST measurements is shown in Table 2, which contains a summary of the results for the optical particle counter analysis:

• Column 1 has the selected particle diameters for the SRM 2806b.
• Column 2 has the IFTS SRM 2806b mean cumulative particle concentrations > the specified column 1 diameters.
• Column 3 has the corresponding IFTS SRM 2806b relative standard deviation (that is, the *k*=1 relative standard uncertainty) for the mean particle concentrations of column 2.
• Column 4 has the NIST SRM 2806b mean cumulative particle concentrations > the specified column 1 diameters.
• Column 5 has the corresponding NIST SRM 2806b relative standard deviation (that is, the *k*=1
relative standard uncertainty) for the mean particle concentrations of column 4.
• Column 6 has the mean particle concentrations > the specified column 1 diameters as specified by the *ad hoc* ISO committee in the *Specification for Manufacturing and Acceptance* criteria. The committee chose the certified values of SRM 2806a to be matched or exceeded. Consequently, the specifications are the certified values of SRM 2806a.
• Column 7 has the specified relative standard deviation (that is, the *k*=1
relative standard uncertainty) for the mean particle concentrations of column 6.
• Column 8 has the measured NIST SRM 2806a mean cumulative particle concentrations > the specified column 1 diameters. Note that these values are informational, showing the optical particle counter was in calibration at the time of the measurements. The SRM 2806a sample was measured by the NIST optical particle counter during this comparison as a verification, and those are the measured values presented here.

**Table 2:** Comparison of the specified particle concentrations to particle concentrations measured by IFTS and NIST for SRM 2806b candidate test material.

Diameter (μm)	IFTS Mean Value (particles/mL)	IFTS RSD (*n*=40)	NIST Mean Value (particles/mL)	NIST RSD (*n*=24)	Specified Mean Values (particles/mL)	Specified RSD	Actual SRM 2806a (particles/mL)
>4	9807	0.0092	7338	0.0097	6095	0.008	6204
>6	3300	0.014	2869	0.012	2395	0.011	2255
>10	799	0.0185	696	0.015	514	0.016	489
>12			401	0.015	281	0.02	268.6
>14	275	0.018	229	0.016	170	0.023	161.3
>20			53.48	0.022	51.35	0.024	52.38
>25	30.18	0.034	25.25	0.028	20	0.045	22.89
>30	15.025	0.044	13.34	0.038	9	0.058	10.51

The systematic differences in the above comparisons are noted and are not understood *per se*, but they are possibly due to differences in sensors used by IFTS and NIST, differences in the calibration of these respective sensors, differences in operators, and differences in measurement protocols.

However, and more importantly, the key data in Table 2 are not in the mean concentrations (columns 2, 4, and 6), but rather the relative standard deviations (columns 3, 5, and 7). For each diameter, it is noteworthy that these relative standard uncertainties: (1) are all comparable, across IFTS 2806b, NIST 2806b, and specified acceptance values, and (2) are all small, which is consistent with the results of the homogeneity and stability analyses. Note that the relative standard uncertainty is not a direct measure of homogeneity and stability, but small relative standard deviations are certainly indicative that any homogeneity and stability issues are minor, with the net result that the SRM is well suited and "fit for purpose."

Table 3 contains the results of the NIST optical particle counter analysis of the clean hydraulic oil with specified levels taken from Table I of the attached specification document in the Appendix. This hydraulic fluid was filtered carefully and sampled before the addition of the ISO medium test dust. By comparison with Table 2, the starting oil is very clean with respect to particles. Recognizing that the small particles are the most difficult to remove, the NIST data indicate that the material is within specification for diameters >4.5 μm. The size calibration of the NIST sensor and the IFTS sensor must be slightly different, since IFTS filtered the material to achieve the cleanliness level required based on their optical particle counter measurements. Although not strictly in agreement with the specified levels as determined by NIST measurements, but within specification by IFTS, the hydraulic fluid was deemed clean enough for purpose.

**Table 3:** Cumulative particle concentration showing level of cleanliness for the hydraulic fluid sample before addition of SAE 5-80 test dust to form SRM 2806b (IFTS data taken from Report of Analysis number 120798, dated April 19, 2012).

Diameter (μm)	Mean IFTS* (particles/mL)	Mean NIST (particles/mL) (*n*=8)	Specified Value (particles/mL)
>4	17.6	392	40
4.2		140	
4.4		49	
4.6		24	
6	5.5	9.2	20
14	0.6	0.84	1.3
20			
21	0.1	0.16	0.32
25	0.08		0.02
30	0.03		
38		0.008	
70		0	0.01

## 8 Study 4: Accepting the Final SRM 2806d Candidate Material

The discussion of Secs. 5, 6, and 7 above has dealt with the IFTS production and NIST testing of SRM 2806b. IFTS produced candidate reference material for SRM 2806b in July 2011 at a nominal particle concentration of RM 8631a of ≈3.5 mg/L. SRM 2806b was released by NIST in June 2014.

IFTS also produced the material for the next-generation dust-in-hydraulic fluid SRM 2806d in July 2018. SRM 2806d was manufactured with approximately 6 mg/L of RM 8631a dust concentration in filtered MIL-PRF-5606 hydraulic fluid. SRM 2806d was placed into sale on March 2021. The analysis of this newer SRM 2806d is the topic of this section.

### 8.1 Bottle Sampling

In July 2018, IFTS produced the hydraulic fluid suspension for SRM 2806d using a test stand with four nozzles (A, B, C, and D). They filled 100 bottles from each nozzle simultaneously (1A, 1B, 1C, 1D), producing a set of 400 bottles labeled 1A to 100A, 1B to 100B, …, 1D to 100D. Bottles analyzed by IFTS and by NIST were chosen by a statistical design. Bottles were taken from the global collection 400 bottles as specified in the Appendix of this document, designed to sample the material over the complete production sequence, but randomized for possible systematic problems like filling nozzle or circulation flow rate variations. As directed in the Appendix, the manufacturer analyzed one bottle out of each 10 bottles produced for within-batch homogeneity testing as described in ISO 11171 clauses F.3 through F.5, except that only 40 bottle samples were analyzed in total. IFTS analyzed bottles in the following series: 10A, 10B, 10C, 10D; 20A, 20B, 20C, 20D, … ; 100A, 100B, 100C, 100D. NIST received and tested eight bottles for within-batch homogeneity using a light blockage automatic particle counter calibrated to the existing SRM 2806b as the calibration material. The eight bottles consisted of two bottles taken from each quartile of the production labeled assignment. The data were compared to analogous data from IFTS. The eight bottles were selected according to the statistical design in the Appendix, and from the 400 bottles, the sample bottles analyzed by NIST were the following (not in this order): 1C, 2C; then 36A, 37A; then 66D, 67D; and finally, 90B, 91B. The NIST bottles were analyzed in a sequence to examine possible manufacturing bias while at the same time removing possible optical particle counter influences.

### 8.2 Statistical Analysis

**Fig. 20:**
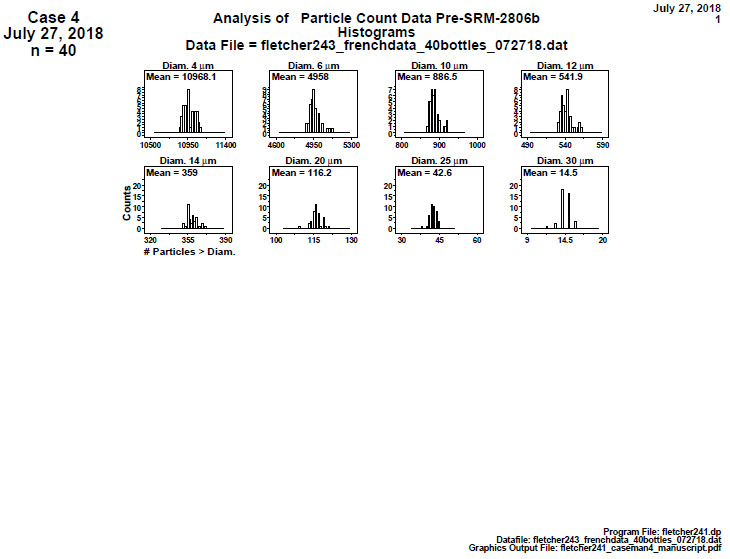
Histogram plots for data collected on 40 bottles by IFTS for SRM 2806d step 1: distributional check: histogram. The vertical axis is the frequency or counts, and the horizontal axis is the cumulative particle concentration > specified diameter; axis labels for all plots are the same as those given on the lower-left plot.

A batch of 400 bottles of candidate SRM 2806d was produced in July 2018. IFTS delivered data, and NIST confirmed their results. A summary of IFTS data and NIST data is shown in the same five-step statistical analysis plots of Fig. 20 to Fig. 24 and Table 4.

#### 8.2.1 Distributional Check: Histograms (Final 2806d)

Conclusion 1: The histograms for the smallest diameter (4) and for the three largest diameters (20, 25, 30) μm are symmetric. The histograms for the "middle" diameters (6, 10, 12, 14) μm are skewed to the right.

#### 8.2.2 Distributional Check: Normal Probability Plots (Final 2806d)

**Fig. 21:**
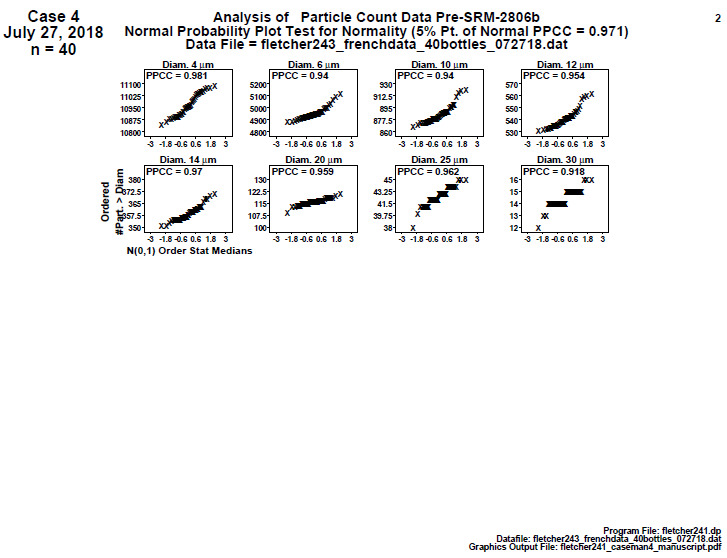
SRM 2806d final material step 2: distributional check: normal probability plots, showing the cumulative particle concentration > specified diameter on the vertical axis and sorted theoretical values for a normal N(0,1) distribution on the horizontal axis. Axis labels for all plots are the same as those given on the lower-left plot.

Note that for *n* = 40 points, the 5% significance cutoff value for the normal PPCC statistic is (as before) 0.972; the 1% significance cutoff value is 0.958.

Conclusion 2: In general, the plots are non-linear (thus non-normal). Only diameter 4 tests out as normal. Diameters 14, 20, and 25 are nonnormal at the 5% level. Diameters 6, 10, 12, and 30 are nonnormal at the 1% level. In light of this non-normality, either the analysis could be redone after a transformation to (and then from) normality, or (in terms of a final estimated certificate reference value) the choice of the (outlier-resistant and transformation-independent) median would be better than the (more outlier-sensitive and skewness-sensitive) mean. The median was in fact used for the consensus value.

#### 8.2.3 Homogeneity and Stability Plots (Final 2806d)

**Fig. 22:**
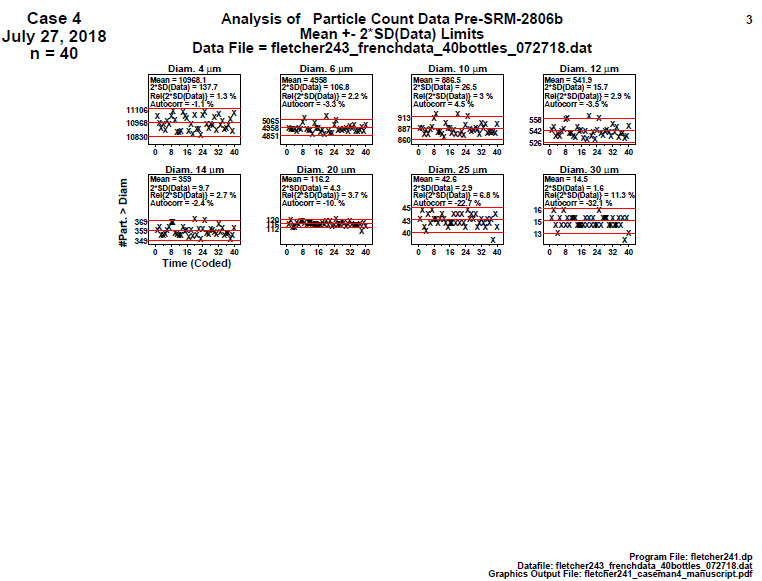
SRM 2806d final material step 3: homogeneity and stability plots showing cumulative particle concentration > specified diameter on the vertical axis and the horizontal axis is the 40 measurements (40 bottles) which are distributed over the approximate 2 h sampling time.

Conclusion 3: For final SRM 2806d analysis, the data have near-zero slope for all eight diameters. This final-stage material manufacturing process thus appears to be quite stable (and predictable) over time. Visually, for any given diameter, the mean of the data for the plot’s second half is approximately the same as the mean of the data for the first half. This plot thus suggests that the manufacturing process is (visually) in statistical control with respect to homogeneity (second half versus first half) and also with respect to stability (near-zero slope and no apparent drifting).

Outlier-wise, the four suspicious values in step 2 for diameters 10 and 12 are showing up as on or outside (above) the (95%) coverage limits for the corresponding step 3 plots. Note that the values are suspicious, but also note that these are 95% coverage limits, and so we would expect 1 in 20 (and 2 in 40) to be outside the limits under perfect conditions, and so the 4 in 40 that we observe is not unusual. For diameter 30, the discreteness of the data is noted. For this largest diameter, the number of distinct response values appears to be small (approximately 5).

#### 8.2.4 Homogeneity and Stability Statistics (Final 2806d)

**Fig. 23:**
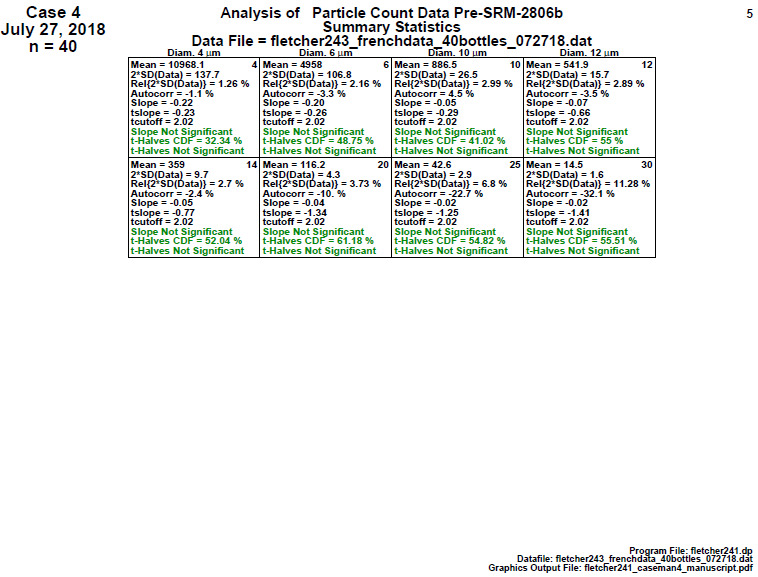
SRM 2806d final material step 4: homogeneity and stability statistics showing 10 statistical results for eight particle diameters.

The most important statistics are shown in the final three lines within each cell, which show the results of a slope (stability) test and a first half versus second half ANOVA (homogeneity) test and its significance. As before, passed tests are in green, indicating statistical insignificance.

Conclusion 4: For all eight diameters, the slope is statistically insignificant, which indicates the process is stable over the 2 h lifetime of the material manufacturing process. Also, for all eight diameters, the ANOVA indicates that both time halves of the process are statistically equivalent (homogeneous). Step 4 thus formally affirms that the material from this final-stage process would well serve as an excellent final candidate material for SRM 2806d.

#### 8.2.5 Homogeneity and Stability Summary Graphics (Final 2806d)

**Fig. 24:**
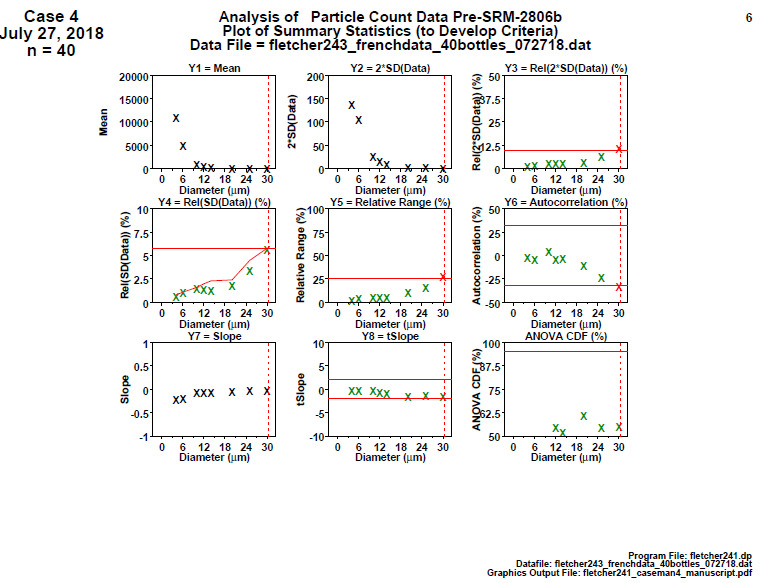
SRM 2806d final material step 5: homogeneity and stability summary graphics. This graphic is a summary of the step 4 information. The vertical axis is the computed statistic, and the horizontal axis is the 30 particle diameters. Quality score is computed to be 3.

Conclusion 5: The last two plots are the most important because they test whether the slope is statistically insignificant (stability) and whether the two halves of the data are statistical equivalent (homogeneity). The SRM 2806d final-stage data are excellent: For all diameters, the slope is statistically flat, indicating a stable process, and the ANOVA is statistically equivalent, indicating a homogeneous process.

The quality score (as indicated by red data points) is 3. The NIST cutoff criterion is 4. This small value is excellent and indicates a stable, homogeneous process in statistical control.

##### 8.2.5.1 Overall Five-Step Analysis Conclusions for this SRM 2806d

Overall conclusions for this SRM 2806d are that the cumulative particle concentration was stable, homogeneous, and invariant and did not depend on when the bottle of test material was produced during the approximate 2 h fill process time. The IFTS work in optimizing their material manufacturing process in combination with the standardized five-step statistical procedures was essential in producing the net result: a delivered set of 400 bottles that constitute the NIST SRM 2806d reference material.

Results for the automatic particle counter analysis are summarized in Table 4, which gives the cumulative particle concentration of SRM 2806d determined by automatic optical particle counter analysis of IFTS and NIST measurements. The cumulative mean particle concentration value for the corresponding particle diameters found in column 1 are shown. IFTS results (*n* =40) are shown in columns 2 and 3. NIST results are in columns 4 and 5 (*n* =24). The specified values suggested by the *ad hoc* ISO committee in the *Specification for Manufacturing and Acceptance Criteria of New SRM 2806d* are shown in column 6 and 7.

**Table 4:** Comparison of the specified cumulative particle concentrations (column 6) to cumulative particle concentrations measured by IFTS (column 2) and NIST (column 4) for candidate SRM 2806d test material.

Diameter [μm(c)]	IFTS Mean Value (1/mL)	IFTS RSD (*n*=40)	NIST Mean Value (1/mL)	NIST RSD (*n*=24)	Specified Mean Values (1/mL)	Specified RSD
> 4	10 968	0.0063	9 433	0.0107	10 000	0.008
> 6	4 958	0.0108	3 962	0.0083	4 200	0.011
> 10	886	0.0150	915	0.0081	1 100	0.016
> 12	542	0.0145	474	0.0082	650	0.02
> 14	359	0.0136	277	0.0079	390	0.023
> 20	116	0.0187	87	0.017	93	0.024
> 25	43	0.033	33.5	0.028	40	0.045
> 30	14	0.0508	13	0.0402	20	0.058

Again, as before, the mean particle concentrations of columns 2, 4, and 6 are less important than the relative standard uncertainties of columns 3, 5, and 7. These relative standard uncertainties are in fact acceptably small (and thus consistent with homogeneity results), and so (even though the new material did not conform perfectly to specification requirements for particle concentration) the *ad hoc* ISO committee in the *Specification for Manufacturing and Acceptance of New SRM 2806* adjudged the material to be suitable, valuable, and fit for addressing community particle counting calibration needs.

Table 5 presents the IFTS results for the clean hydraulic fluid, filter processed in their test stand, and bottled suspension for reference testing. The cumulative particle concentration values are well within specification.

**Table 5:** Particle concentrations showing level of cleanliness for the filtered hydraulic fluid used to make SRM 2806d before addition of ISO medium test dust (data taken from the IFTS Report of Analysis 182317, December 2018).

Diameter (μm)	Mean (1/mL) (*n*=8)	Mean On-Line (1/mL)	Specified Value (1/mL)
>4	10.8	8.6	40
6	4.2	1.8	20
10	1.2	0.32	
12	0.8	0.16	
14	0.6	0.12	1.3
20	0.3	0.08	
21		0.08	0.32
25	0.1	0.04	
30		0	0.05

## 9 Summary and Conclusions

By working together, IFTS and NIST were able to improve a process valuable to important industrial sectors by producing a high-quality reference material with which to standardize their measurements. An acceptance methodology was developed that tested the quality of two NIST SRM 2806 materials, SRMs 2806b and 2806d, and this method can be used in the future for any next-generation materials. The metrology entailed particle measurements using optical particle counters to make independent measurements by both the manufacturer and by NIST and comparison of results. Also, a subset of the material, if it passed certain criteria, was sent to NIST, where the material was examined by SEM and subjected to an accelerated aging test to verify the fluid was stable. The aged material was compared by optical particle counter and SEM measurements to materials not subjected to harsh environments. IFTS and NIST worked together to produce a medium test dust in hydraulic fluid SRM that was acceptable for NIST certification and of high quality for the user community.

A key tool for both IFTS and NIST in the development of SRMs 2806b and 2806d was a five-step statistical graphics analysis methodology that was used to assess homogeneity and stability of the candidate material production process. This presented EDA methodology was applied to all results submitted to NIST and applied by the material manufacturer to test in-house the quality of their material under production. This paper presented four separate SRM-related material analysis examples that illustrate the utility of the methodology. The first analysis example involved an early-stage (Dec. 2010) on-line IFTS effort in the SRM 2806b material production process. The five-step diagnostic process indicated that this early material was not homogeneous or stable enough for the NIST SRM; inhomogeneity and down-drift issues existed from the beginning of the manufacturing process to the end.

Study 2 (July 2011) of on-line particle data showed that the candidate material was markedly improved; this improvement was at least in part enabled by IFTS implementing and utilizing the five-step software (DATAPLOT) tool on-site and on-line in their production process to achieve real-time feedback on production modification efforts. The net value added was that resulting material was in fact considerably improved (homogeneous and stable) and closer to NIST reference material acceptance.

Study 3 (July 2011) involved the analysis relevant to SRM 2806b. This analysis was on manufactured bottles of material and consequently was very important for the acceptance of the new material for certification. Because the material passed the process-control statistical tests and because the bottle-to-bottle relative standard uncertainties were satisfactorily small, NIST accepted the material and used it to produce SRM 2806b.

Study 4 (July 2018) involved SRM 2806d bottled material, for which the five-step diagnostics highlighted the existence of some homogeneity and stability issues, but the small relative standard uncertainties and overall quality resulted in the appropriate ISO committee deeming the material suitable for use by the general particle counting industrial community.

The analysis results shown here for both SRM 2806b and SRM 2806d led to both materials being accepted by NIST and subsequently certified. SRM 2806b was certified by microscopy and image analysis made traceable to the NIST line scan primary standard [9], and SRM 2806d was certified by an interlaboratory study containing 22 measurement results from 13 national and international participants [10]. The traceable link was through SRM 2806b, which was used as the calibrant both directly and indirectly through a secondary standard. The IFTS and NIST collaboration produced a medium test dust in hydraulic fluid SRM that was NIST certified and of high quality for the user community. The approach outlined in this paper can be used for future SRM 2806x materials, to ensure the industrial and military customers are provided with a needed certified reference material.

## 10 Appendix

The appendix was formulated by NIST and members of the ISO committee. It was never published, nor formally documented in any publication. It was used to guide producers of the material and will be used in some form in the future to facilitate the production of new SRM 2806. Provided here as reference.

### 10.1 Specifications for Manufacturing and Acceptance of New SRM 2806 Material D**ated: January 12, 2017**

Standard Reference Material 2806 is composed of medium test dust suspended in filtered hydraulic fluid. A minimum of 400 bottles of this material shall be produced under the specifications outlined below.

#### 10.1.1

Production Method

Prepare samples according to Annex F of ISO 11171 with the following specifications (pertinent clauses are indicated):

Produce a minimum of 400 bottles (clause F.2),

Total volume of suspension to be at least 200 L (clause F.2),

*10.1* Before addition of dust, oil in the sump cleaned to a degree such that the oil contains fewer than the maximum allowable contaminant levels shown in Table I for particle sizes larger than 4, 6, 14, 21, and 30 µm(c), respectively (clause F.2).

*10.2* Dust concentration must fall between 10 000 particles/mL and 17 000 particles/mL larger than 4 μm(c) and between 20 particles/mL and 300 particles/mL larger than 30 μm(c). Particle sizes to be analyzed include, but are not limited to, (4, 6, 10, 12, 14, 20, 25, and 30) µm(c) (clause F.5).

*10.3* On-line particle sensor installed on the primary recirculation line.

*10.4* Batch and process to be validated according to acceptance criteria described below, instead of clause F.5.

*10.5* Particulate material is RM 8631, which will be supplied by NIST to the manufacturer.

**Table I:** Maximum allowable contaminant levels for clean fluid and bottles.

Particle Size (µm(c))	Concentration of particles (particles/mL) Larger than Indicated Size
4	40
6	20
14	1.3
21	0.32
30	0.05

#### 10.1.2

Single Batch of Material

A minimum of 400 bottles shall be produced from the same sump of hydraulic fluid medium–test dust suspension. From these 400 bottles, 40 bottles will be used by supplier to demonstrate bottle-to-bottle homogeneity at a level of acceptance that will be provided by NIST, 24 bottles are to be sent to and used by NIST for determining acceptability, and 10 bottles are to be held in reserve by supplier for reference purposes.

#### 10.1.3

Bottle

Each bottle shall contain 400 mL of hydraulic fluid medium–test dust suspension. Bottle caps are to be made of a hard phenolic material, and bottle cap liner or inner seal should be made of Teflon. The seal shall be attached to the cap and washed with filtered ethanol or isopropyl alcohol. The seal and cap must be completely dry, void of all liquid, before it is placed on the bottle.

#### 10.1.4

Bottle Requirements

Bottles are to be precleaned of particles to a level cleaner than the maximum allowable contaminant levels shown in Table I for particles larger than (4, 6, 14, 21, and 30) μm(c), respectively (clause F.2). The sample bottles shall be cleaned and verified in accordance with ISO 3722 according to the sampling scheme defined by NIST. Each bottle will be labeled according to the scheme *X*-*Y*-*Z*, where *X* refers to the batch number assigned by NIST, *Y* refers to the order in which the bottle was filled (*e.g.*, *Y*=1 for the first bottle, *Y*=2 for the second, *etc*.), and *Z* refers to a letter designating the sampling port used to fill the bottle. The labels are to be applied to the corresponding bottle cap and must not include any company references, logos, or names. No label is to be applied or attached directly to the bottle itself.

#### 10.1.5

Particulate Material

NIST RM 8631 will be provided by NIST within 30 calendar days after contract award.

#### 10.1.6

Hydraulic Oil

Exxon Mobil MIL-PRF-5606 hydraulic fluid will be used.

#### 10.1.7

Particle Concentration

Particle concentration must fall between 10 000 particles/mL and 17 000 particles/mL larger than 4 μm(c) and between 20 particles/mL to 300 particles/mL larger than 30 μm(c).

#### 10.1.8

Additive to the Oil

Stadis 450 antistatic additive should be added to adjust the conductivity of the oil to 2500 ± 250 pS/m.

#### 10.1.9

Acceptance Criteria

The manufacturer will analyze one, randomly-chosen bottle out of each 10 bottles produced for within-batch homogeneity as described in ISO 11171:2016 clauses F.3 through F.5, except that only a total of forty (40) bottle samples will be analyzed. For example, one randomly chosen bottle will be chosen from the first 10 collected (bottles 1–10), one from the second group of 10 bottles (bottles 11–20), one from the third group of 10 bottles (bottles 21–30), *etc*. This is a reduction from the requirements of ISO 11171 (every fifth sample) because an additional requirement is specified to conduct continuous on-line automatic particle counts to verify the uniformity of the suspension being prepared.

The results must meet the acceptance criteria specified in clause F.5 and using the allowable coefficient of variation (CoV) specified in Table II The particle count results shall be provided to NIST for verification. If the results for all sizes do not meet these acceptance criteria, or if they show evidence of a systematic bias, then the batch will be rejected.

Lack of systematic bias in the manufacturing cycle will be verified first by the manufacturer using a set of NIST-generated statistical tests. NIST will provide a software package to be used by the manufacturer that will contain the following tests: mean, standard deviation, slope of the sample time sequence, autocorrelation, and an ANOVA test. These tests will help the manufacturer ensure that there is no bias in their process. *Data used in these tests and the results will be sent to NIST.*Acceptance boundaries are built into the software. Once these boundaries are met, the next step will require shipment of select bottles for NIST analysis (9.4).

NIST will receive and test eight bottles for within-batch homogeneity using a light blockage automatic particle counter calibrated according to ISO 11171 using SRM 2806. The eight bottles will consist of two bottles taken from each quartile of the production labeled assignment. The data will be compared to an analogous set of eight bottles analyzed by the manufacturer. The eight bottles from the manufacturer will be selected from among those samples previously analyzed to qualify the batch according to acceptance criteria given in ISO 11171 clauses F.3 through F.5 and will consist of those samples sequentially nearest to the bottles selected for NIST analysis. For example, if there are 400 bottles, the sample bottles to be analyzed by NIST would be the following: 98, 99; then 198, 199; then 298, 299; and finally, 398, 399. The data from these bottles would be compared to the data from bottles 90, 100; 190, 200; 290, 300; and 390 and 400 analyzed by the manufacturer. Both NIST and the manufacturer’s results must show CoV values less than or equal to the values found in Table II and there must be no evidence of systematic bias. In counting each bottle, at least three counts per bottle must be obtained, and at least 240 mL from each bottle must be counted. The particle count results by both NIST and the manufacturer shall be documented. If data from NIST and supplier for the specified 16 samples (8 analyzed by NIST and 8 analyzed by the manufacturer) do not agree at all specified sizes, then the batch will be rejected.

**Table II:** Values of CoV not to exceed for particle diameter determined by SRM 2806.

Particle Size (µm(c))	CoV (Fraction)^a^
4	.008
6	.011
10	.016
12	.020
14	.023
20	.034
25	.045
30	.058

a Note: These are for cumulative counts for eight bottles and three counts each (*n*=24). These values for CoV have been confirmed to be achievable using existing sample production techniques.

The manufacturer shall analyze the on-line particle count data collected by calculating the mean, standard deviation, and CoV for each particle size. The resulting CoV for each particle size must be less than the acceptable limits shown in Table II.

All samples tested will exhibit a stable size distribution for a time period of 3 months at normal storage temperatures and conditions (~22 °C) and after 3 months of accelerated aging, during which samples are stored at temperatures of 75 °C and 12 °C (all tests will run simultaneously). Size distributions are to be verified by NIST using automatic particle counting and scanning electron microscopy.

The mean of the cumulative concentration found from the 8 bottles for the designated particle diameters for both NIST and the manufacturer must be greater than the lower acceptable concentration found in Table III.

**Table III:** Lower particle acceptable concentrations pertaining to mean values for the 8 test bottles.

Particle Size [µm (c)]	Lower Acceptable Concentration^a^ (particles/mL Larger than Indicated Size)
4	10 000
6	4 200
10	1 100
12	650
14	390
20	93
25	40
30	20

a These values were determined from the NIST certification for SRM 2806b and are set to ensure that adequate particles are present for calibration at larger particle sizes.

#### 10.1.10

To Be Delivered upon Meeting Acceptance Criteria

• The remaining 326+ bottles of the manufactured batch of medium test dust in hydraulic fluid.

• Twelve (12) additional bottles of hydraulic fluid identical to those produced as described in acceptance criteria 1, except that these were collected immediately prior to the addition of test dust to the oil. These will be used to assess contamination in the test fluid. All of the sampling ports used to produce the 326+ bottles shall be used to produce these 12; *i.e.*, if four sampling ports were used, then three of these clean oil samples shall be taken from each of the four ports.
• Six (6) clean bottles with closures, cleaned in the same manner as those used to collect the samples.
• Relevant physical/chemical specifications for the batch material, including Material Safety Data Sheet, the electrical conductivity, viscosity, density, additive concentration, oil and additive specifics (source, manufacturer, grade, batch number, *etc*., possibly oil analysis results such as spectroscopy and/or chromatography), and concentrations of the following elements: Ag, Al, B, Ba, Ca, Cd, Cr, Cu, Fe, K, Mg, Mn, Mo, Na, Ni, P, Pb, Sb, Si, Sn, Sr, Ti, Vd, and Zn. Additional physical and chemical data on the material to be provided as requested.

#### 10.1.11

Delivery Milestones

Required contract completion date is 180 calendar days after award. Shown below (Table IV) are desirable and approximate guidelines for performance. All days are calendar days. The process can proceed faster or slower depending upon the manufacturing process control.

**Table IV:** Delivery milestones.

Step	Due Date	Action
1	0 d	Contract awarded.
2	60 d after award	Complete the following: (1) Produce 400 bottles of candidate SRM 2806 as per specification, (2) collect on-line particle counts, and (3) collect particle counts from 40 bottles. Test data with NIST-provided statistical software and provide all raw particle counts and statistical test results to NIST by email.
3	75 d	NIST reviews data and may have questions.
4		If data are within specifications, NIST will request shipment of select bottles from the batch. If data do not meet specification, return to step 2.
5	90 d	Company ships only requested bottles from the production batch. *Do not ship the entire batch.*
6	90 d concurrent with step 5	NIST performs stability testing on product sent in step 5 and discussed in 9.6 above.
7	120 d	NIST completes analysis on selected bottles and compares and shares results with company.
8	150 d	Company emails final report including all data requested in the specification.
9	170 d	After NIST review of report and only upon NIST request, company ships the remaining bottles of hydraulic fluid and other items requested in specification.
10	175 d	NIST receives shipment, checks for damaged bottles, and analyzes select bottles.
11	180 d	NIST reviews material stability test results and confirms material has not altered.
12	180 d	Contract completed.
